# Dynamic transcriptome analysis suggests the key genes regulating seed development and filling in Tartary buckwheat (*Fagopyrum tataricum* Garetn.)

**DOI:** 10.3389/fgene.2022.990412

**Published:** 2022-08-22

**Authors:** Liangzhen Jiang, Changying Liu, Yu Fan, Qi Wu, Xueling Ye, Qiang Li, Yan Wan, Yanxia Sun, Liang Zou, Dabing Xiang, Zhibin Lv

**Affiliations:** ^1^ Key Laboratory of Coarse Cereal Processing, Ministry of Agriculture and Rural Affairs, Sichuan Engineering & Technology Research Center of Coarse Cereal Industralization, College of Food and Biological Engineering, Chengdu University, Chengdu, China; ^2^ College of Tourism and Culture Industry, Chengdu University, Chengdu, China; ^3^ Department of Medical Instruments and Information, College of Biomedical Engineering, Sichuan University, Chengdu, China

**Keywords:** Tartary buckwheat, seed development, transcriptome, expression analysis, expansin, phytohormone pathways, starch biosynthesis

## Abstract

Tartary buckwheat is highly attractive for the richness of nutrients and quality, yet post-embryonic seed abortion greatly halts the yield. Seed development is crucial for determining grain yield, whereas the molecular basis and regulatory network of Tartary buckwheat seed development and filling is not well understood at present. Here, we assessed the transcriptional dynamics of filling stage Tartary buckwheat seeds at three developmental stages by RNA sequencing. Among the 4249 differentially expressed genes (DEGs), genes related to seed development were identified. Specifically, 88 phytohormone biosynthesis signaling genes, 309 TFs, and 16 expansin genes participating in cell enlargement, 37 structural genes involved in starch biosynthesis represented significant variation and were candidate key seed development genes. *Cis*-element enrichment analysis indicated that the promoters of differentially expressed expansin genes and starch biosynthesis genes are rich of hormone-responsive (ABA-, AUX-, ET-, and JA-), and seed growth-related (MYB, MYC and WRKY) binding sites. The expansin DEGs showed strong correlations with DEGs in phytohormone pathways and transcription factors (TFs). In total, phytohormone ABA, AUX, ET, BR and CTK, and related TFs could substantially regulate seed development in Tartary buckwheat through targeting downstream expansin genes and structural starch biosynthetic genes. This transcriptome data could provide a theoretical basis for improving yield of Tartary buckwheat.

## 1 Introduction

Seed is the most important organ of crop. Seed development is an essential and complex process, which involves seed size change and nutrients accumulation (Kong et al., 2015). Broadly, seed development can be divided into two important phases: embryogenesis and maturation. In embryogenesis phase, cells divide and expand to establish the tissues and organelles, while in the maturation phase, resources are allocated to synthesize storage compounds (Ruuska et al., 2002). Kernel weight of seeds plays a vital role in the yield of cereal crops and is determined by the duration and rate of grain filling. Thus, improving grain filling increases the grain weight and cereal yield (Savadi, 2018). Grain filling is predominantly regulated by genetic factors and is also greatly influenced by physiological pathways and environmental factors. Phytohormones, including auxin (AUX), cytokinin (CTK), gibberellin (GA), brassinosteroid (BR), abscisic acid (ABA), and ethylene (ET) contribute to seed development (Jameson and Song, 2016; Savadi, 2018; Li N. et al., 2019; Kozaki and Aoyanagi, 2022). Various genes involved in different mechanisms control the process of seed development/grain filling (Tzafrir et al., 2004). Recently, much research was conducted to investigate the specific genes or pathways controlling seed development in barley (Bian et al., 2019), rice (Bian et al., 2019), maize (Chen et al., 2014; Li et al., 2014), wheat (Cantu et al., 2011; Li et al., 2013), soybean (Jones and Vodkin, 2013; Lu et al., 2016; Du et al., 2017), chickpea (Garg et al., 2017), and *Brassica napus* (Basnet et al., 2013; Zhou et al., 2017). These genes include TFs, hormone related genes, genes involved in seed size regulation, seed storage proteins (SSPs), starch and lipid biosynthesis. However, key seed developmental genes and their regulatory networks in plants were far more than elucidated, especially in those non-model crops.

Tartary buckwheat (*Fagopyrum tataricum* Garetn.), as an edible and medicinal non-model crop, is becoming highly attractive for the high-quality proteins and pharmaceutical ingredients, such as flavonoids, polyphenols, and D-chiro-inositol in the seeds (Li and Zhang, 2001). However, the yield of Tartary buckwheat is only about 1500 kg/ha, which is remarkably lower than staple crops, such as rice or wheat. The production is hard to be broken-through by laborious agricultural strategies only (Xiang et al., 2016; Xiang et al., 2019; Xiang et al., 2020). Seed development is crucial for determining grain yield, and elucidation of the molecular mechanism of seed development could potentially improve yield through molecular breeding. Nevertheless, poor post-embryonic grain filling always occurred in Tartary buckwheat seed development process, which greatly hinders the grain yield improvement. Therefore, it is of considerable interest to identify key genes and dissect the molecular mechanisms of seed development in Tartary buckwheat.

Recently, *FtARF2* was reported to promote Tartary buckwheat fruit enlargement by prolonging the cycle of embryonic development and increasing the cycle of cell division (Liu et al., 2018b). Cytochrome P450 monooxygenase superfamily participating in the synthesis of flavonoids, plant growth and development in Tartary buckwheat were clarified (Sun et al., 2020). Five members of *FtCYP78A* family were suggested to be candidate genes that regulate seed size (Sun et al., 2020). Other studies of the Tartary buckwheat seed development by transcriptome analysis mainly focused on the molecular foundation of nutrients accumulation, such as flavonoid (Gao et al., 2017; Huang et al., 2017; Liu et al., 2018a; Li H. Y. et al., 2019). Yet other mechanisms involved in the molecular basis and regulatory network of seed development, especially those governing post-embryonic grain filling process, such as cell enlargement and starch accumulation, were not clarified. Thus, we carried out global transcriptional expression profiling at three stages spanning important developmental stages of seed development to identify the potential regulators involved in these processes in Tartary buckwheat seeds.

## 2 Materials and methods

### 2.1 Plant materials and growth conditions

Seeds of Tatary buckwheat (*F. tataricum* cv. Xiqiao No.1) was used as the experimental materials in this study, which were obtained from the Key Laboratory of Coarse Cereal Processing, Ministry of Agriculture and Rural Affairs, Chengdu, Sichuan Province, China. The seeds were sown and grown in plastic pots (25 cm in diameter, 20 cm in height) at the density of 8 seeds for each pot, and the seedlings were thinned to three at the early vegetative stage (cotyledons). Each pot contained 15 kg of air-dried soil (remove large stones, plant roots, and other litter), and the soil was sandy soil in texture and alkaline (pH = 7.88) with 48.3, 20.7, and 31.1 mg kg^−1^ available N, P, and K, respectively; 0.76, 0.49, and 12.8 g kg^−1^ total N, P, and K, respectively; and 10.2 g kg^−1^ organic matter. The soil physical properties (0–0.2 m) were determined according to the method proposed by Pang et al. (2006). The pot was placed in the field to keep consistent with field production under normal agricultural management.

**FIGURE 1:**
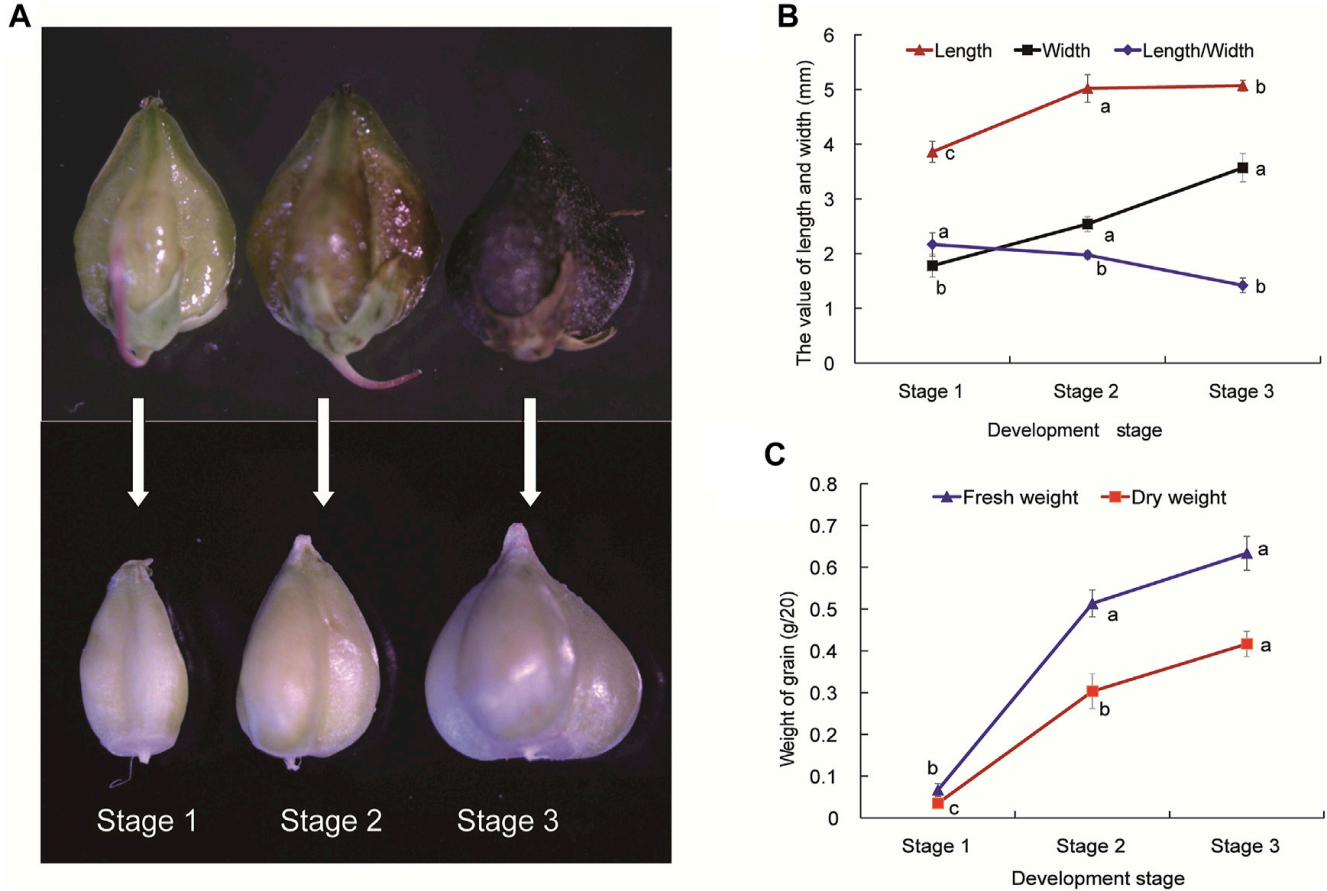
The developmental stages **(A)**, agronomic traits **(B)** and weight **(C)** of Tartary buckwheat seeds. Stage 1 (S1), Seed formation started, torpedo embryo; Stage 2 (S2), Milk-ripe stage, the endosperm is solidifying, initial mature embryo; Stage 3 (S3), The seed matured, mature embryo and pericarp was totally black. Data are presented as the mean ± SD of three biological replicates. Grains were divided into different developmental stages with 20 grains measured at each stage. Different letters denote significant differences (*p* < 0.05).

At the beginning of anthesis stage, we labeled and recorded the time of flowering, as to determine the time of anthesis and developmental stage of grain. The time of anthesis and developmental stage of Tartary buckwheat grain were determined as previously described (Song et al., 2016). We selected the grain of Tartary buckwheat at the stage 1, stage 2 and stage 3 (Stage 1, Seed formation started; Stage 2, Milk-ripe stage, the endosperm is solidifying; Stage 3, The seeds matured, and pericarp was totally black; Figure 1) to collect the sample, and three biological replicates were sampled. For each sample, seeds were collected directly into liquid nitrogen by decorticating the grain from 20 to 30 individual plants for the purposes of homogeneity.

### 2.2 Analysis of seed morphology and weight

The twenty grains per replicate were sampled and measured at stage 1, 2 and 3 (Figure 1), and three biological replicates were sampled. The length, width and length/width of grain, fresh and dry weight of grain were measured. The dry weight was oven-dried at 65°C to constant weight and then measured.

### 2.3 Sample collection and preparation for RNA sequencing

The purity, concentration and integrity of RNA samples are tested using advanced molecular biology equipment to ensure the use of qualified samples for transcriptome sequencing. A total amount of 1 μg RNA per sample was used as input material for the RNA sample preparations. Sequencing libraries were generated using NEBNext UltraTM RNA Library Prep Kit from Illumina (NEB, United States) following the manufacturer’s recommendations and index codes were added to attribute sequences to each sample.

The clustering of the index-coded samples was performed on a cBot Cluster Generation System using TruSeq PE Cluster Kit v4-cBot-HS (Illumia) according to the manufacturer’s instructions. After cluster generation, the library preparations were sequenced on an Illumina platform and paired-end reads were generated.

### 2.4 Data analysis

The adaptor sequences and low-quality sequence reads were removed from the data sets. Raw sequences were transformed into clean reads after data processing. These clean reads were then mapped to the reference genome sequence of the Tartary buckwheat (Pinku1) genome (http://www.mbkbase.org/Pinku1/) using TopHat (v2.0.12) (Zhang et al., 2017). Only reads with a perfect match or one mismatch were further analyzed and annotated based on the reference genome. Hisat2 tools soft were used to map with reference genome.

Differential expression analysis of two conditions/groups was performed using the DEseq. DEseq provide statistical routines for determining differential expression in digital gene expression data using a model based on the negative binomial distribution. The resulting *p* values were adjusted using the Benjamini and Hochberg’s approach for controlling the false discovery rate. Genes with an adjusted *p*-value < 0.01 found by DEseq were assigned as differentially expressed. To gain insight into the function of DEGs, KEGG enrichment analysis of the DEGs was implemented by using the KOBAS software (Kanehisa et al., 2008). Pathways with correlated *p* value less than 0.05 were assigned as significantly enriched items.

K-means clustering analysis of DEGs using the k-means function in R, where k = 12 within the cluster package by Euclidean distance. Heat maps were drawn using the P heatmap package in R and were clustered using Pearson correlation distance. The search for orthologous genes of seed size-related genes from other species in Tartary buckwheat (Pinku1) genome was performed *via* TB tools (Chen et al., 2020). The promoter sequences (about 1.5k bps upstream of the transcription start site) of DEGs were extracted from Tartary buckwheat (Pinku1) genome by TB tools. The *cis*-regulatory elements enrichment analysis of the promoter area of DEGs involved in seed development was performed by the online prediction tool Plantcare (Lescot et al., 2002) (http://bioinformatics.psb.ugent.be/webtools/plantcare/html/), with defaulted parameters for TF family assignment and thresholds. The illustration of the predicting TF-binding sites was drawn by TB tools.

## 3 Results

### 3.1 Shape and weight change of seeds during Tartary buckwheat development

To explore the molecular mechanisms of grain filling process during Tartary buckwheat seed development, three stages (Stage 1, Seed formation started, torpedo embryo; Stage 2, Milk-ripe stage, initial mature embryo, the endosperm is solidifying; Stage 3, The seeds matured, mature embryo, the pericarp was totally black; Figure 1A) of seeds were selected. Several agronomic traits including length, width, and length/width, fresh and dry weight were measured. As shown in Figure 1A, outer fruit shape changed gently with the seed development (upper picture), with both the length and width of seed coat reaching a maximum size in S3 phase, which sets an upper limit to final size of a grain. Yet significant sharper physiological changes were seen among the dehulling seeds without coats, which continuously expanded until the maturation phase in Tartary buckwheat (Figure 1A, lower picture). As kernel weight of seeds plays a vital role in the yield of cereal crops, dehulling seeds were then analyzed for the grain filling process in the following research. The seed length and width, especially width, increased significantly (*p* < 0.05) from stage 1 to stage 3 (Figure 1B). The ratio of seed length to width was substantially the highest at stage 1 among three stages, and the grains became conical gradually after stage 2. The weight of grains changed significantly (*p* < 0.05) during seed maturation (Figure 1C). Accompany with the shape change, both the fresh and dry weight of Tartary buckwheat seed considerably elevated with the seed growth, with the highest in stage 3 (0.63 and 0.42 g/20 grains, respectively).

### 3.2 Transcriptional profiles of filling stage seeds

The dynamics of mRNA abundance at three pivotal stages (Figure 1) of grain development in Tartary buckwheat were assessed. Totally, 40.67 Gb of raw data was obtained for all of the samples, with the average about 22.6 million pair-end reads with 150 bp in size for each sample. After removing the low-quality reads and adaptor sequences from reads, in total we obtain 203 million clean reads from S1, S2 and S3, which yielded 7.16 billion, 6.46 billion and 6.63 billion nucleotides, respectively (Table 1). These clean reads could represent 94.07% of the raw data. With respect to GC content, the S1, S2 and S3 library reached 48.78, 47.55 and 46.23%, respectively (Table 1). After quality filtering, all of the clean reads were mapped to the reference genome of Tartary buckwheat (Zhang et al., 2017). The results showed that on average 45.19 million paired-end reads (93.59%) could be mapped to the reference genome. The average number uniquely mapped to the reference genome at three stages was 39.70 million paired-end reads (87.77%), with the range from 38.26 (88.54%) to 40.03 (90.18%) (Table 1).

**TABLE 1:** Characteristics of generated read data and results of sequence mapped to the reference genome.

Item	Grain development stage		
	S1	S2	S3
Total Reads	47,963,131	43,215,621	44,398,329
Clean reads	23,981,565	21,607,810	22,199,164
Clean base number	7,163,455,810	6,457,976,261	6,631,570,818
GC content (%)	48.78	47.55	46.23
Q30 percentage (%)	94.21	93.57	94.42
Mapped Reads	44,285,248 (92.09%)	40,878,284 (94.59%)	41,774,374 (94.10%)
Uniq Mapped Reads	40,802,372 (84.60%)	38,259,936 (88.54%)	40,027,825 (90.18%)
Multiple Map Reads	3,482,875 (7.49%)	2,618,348 (6.05%)	1,746,549 (3.92%)
Reads Map to “+”	21,874,547 (45.46%)	20,157,291 (46.65%)	20,732,040 (46.70%)
Reads Map to “−”	21,977,813 (45.68%)	20,322,772 (47.03%)	20,779,812 (46.81%)

**FIGURE 2:**
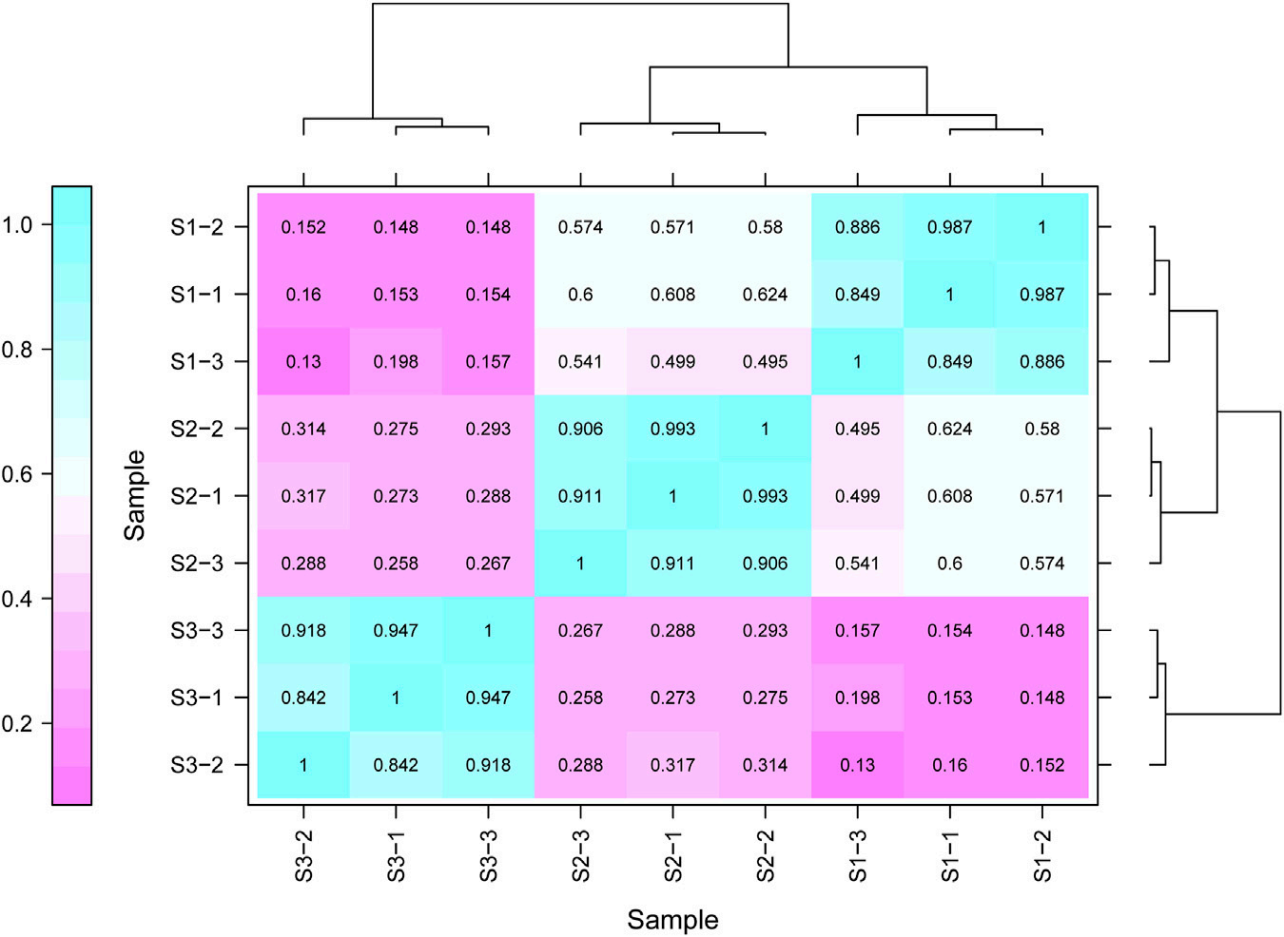
The correlation data sets between the gene expression and three growth stages of Tartary buckwheat with three biological duplicates.

**FIGURE 3:**
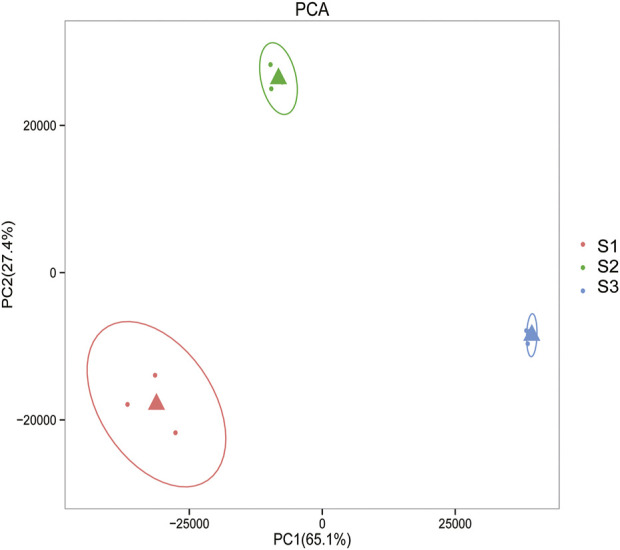
Principal component analysis of gene expression profiles indicated that all the samples were divided into three distinct groups.

**FIGURE 4:**
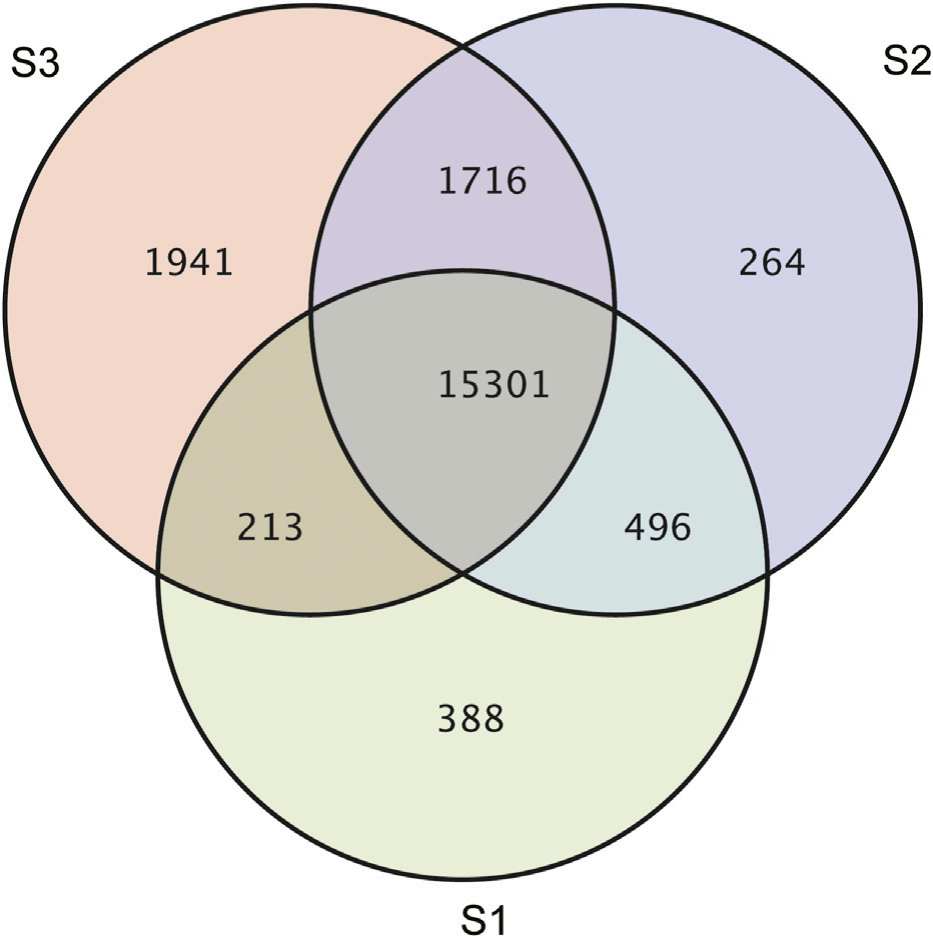
A Venn diagram showing the specifically or commonly expressed genes in different groups.

Gene expression pattern was calculated by the fragments per kilobase of exon per million mapped reads (FPKM) method. Based on the gene expression levels, Pearson correlation coefficient between different samples was calculated. The results indicated that three biological replicates of all samples demonstrated consistent determinations of transcript abundance with a coefficient (R^2^) greater than 0.842 (Figure 2). Simultaneously, the correlation between gene expression and different developmental stages was compared. We found that the coefficients of S1 and S2 were 0.624 (R^2^ < 0.63), while those of S2 and S3 were even lower, with the value <0.317. PCA (Principle Component Analysis) grouping of different sample expression profiles displayed that all the samples were separated into three groups, which is consistent with the correlation results above (Figure 3). Venn diagram analysis showed that 15,301 genes were ubiquitously expressed in all samples, and 388, 264 and 1,941 genes showed specific expression in S1, S2 and S3, respectively (Figure 4). S3 harbored more distinctly-expressed genes than S2 and S1.

### 3.3 Analysis of differentially expressed genes among the seeds at three developmental stages

**FIGURE 5:**
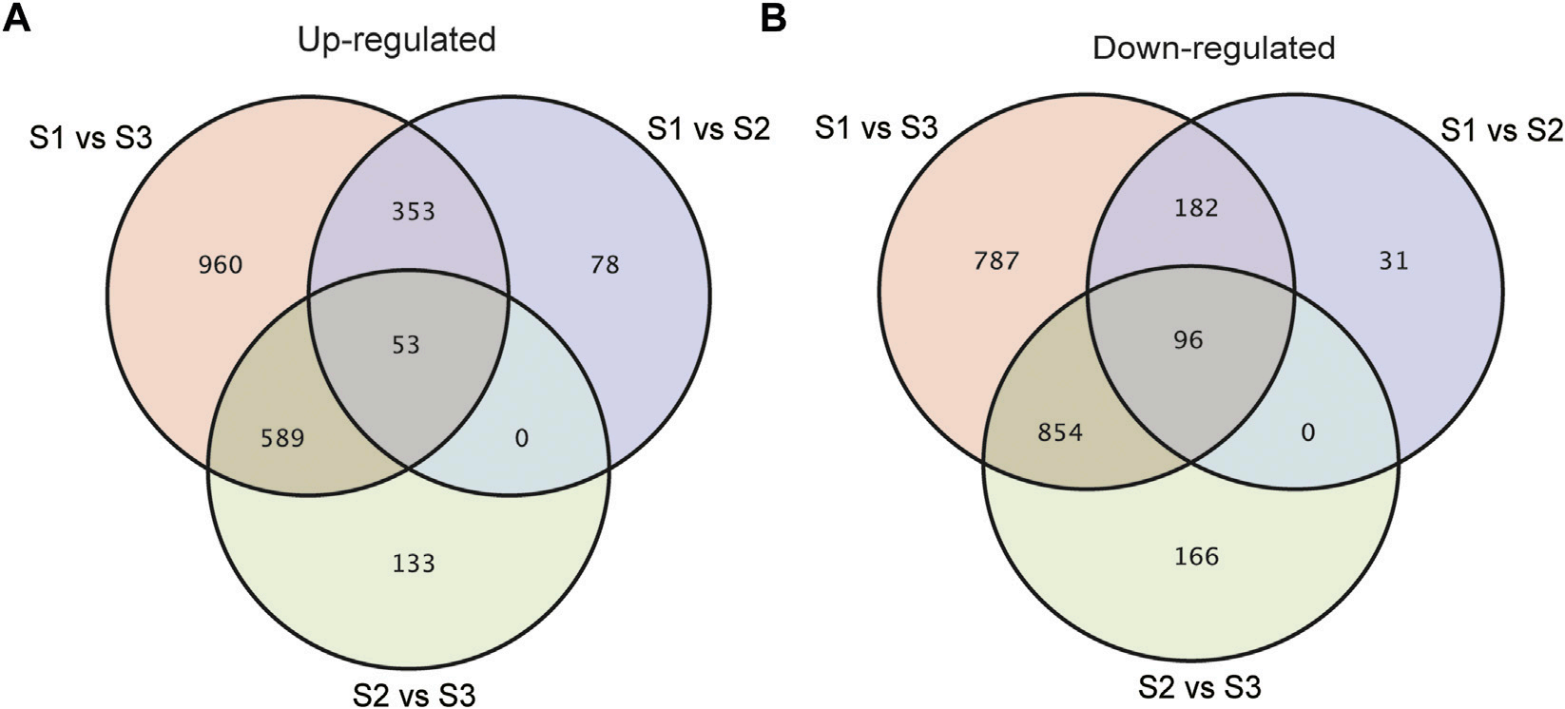
Venn diagrams showing the up-regulated **(A)** or down-regulated **(B)** DEGs in pair-wised comparison groups with *p* < 0.01 and FC > 4.

With an adjusted *p*-value (P) < 0.01 and fold change (FC) > 2, 10065 significant differentially expressed genes (DEGs) were identified totally at three stages of seed development. As shown in Supplementary Figure S1, compared with S1, 1771 and 1330 genes increased and decreased the expression respectively in S2, while the number of genes being up-regulated and down-regulated specifically in S3 was 4500 and 4135 respectively (Supplementary Figure S1). To acquire the information of key genes involved in seed filling/development in Tartary buckwheat, a cutoff of FC > 4 and *p* < 0.01 was applied for gene analysis. In brief, a total of 4249 DEGs were identified by pair-wised comparison of three stages of seed development, among which 376 were new genes. These 4249 DEGs were selected for subsequent further analysis. 793, 1891 and 3874 genes were differentially expressed in comparison of S1 vs. S2, S2 vs. S3 and S1 vs. S3, respectively (Supplementary Figure S2). Compared with S1, 406 and 278 DEGs were commonly up-regulated and down-regulated respectively both in S2 and S3 (Figures 5A,B). Moreover, 78 and 31 DEGs were specifically up-regulated and down-regulated in S2 respectively by comparing with S1, while 1549 and 1641 DEGs respectively were specifically up-regulated and down-regulated in S3. 133 up-regulated genes and 166 down-regulated genes respectively were identified particularly in comparison S2 vs. S3. This together with the PCA result indicated that sharp transitions in gene expression occurred along with the seed development in Tartary buckwheat, especially in the transition from S2 to S3, which is consistent with the shape and weight changes of seeds in Figure 1 and previous reports that significant transcriptional and physiological changes occurred with seed growth in Tartary buckwheat (Liu et al., 2018a).

**FIGURE 6:**
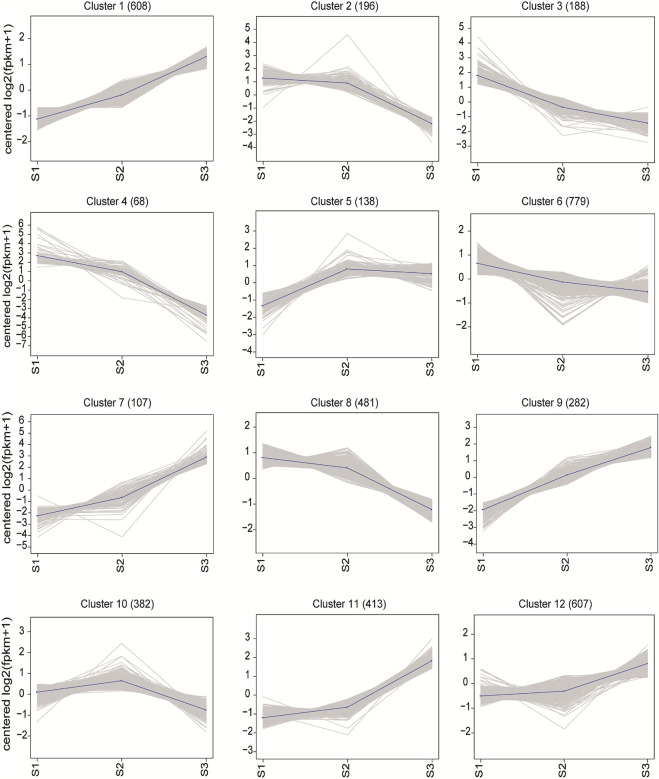
Clusters of co-expressed genes and their kinetic patterns during the seed development.

To disclosure the expression pattern of the DEGs during seed development, the gene expression profile clustering was conducted by the K-means method. All DEGs were assigned to 12 different kinetic clusters with similar expression patterns (Figure 6). Though with different extent of variation, Cluster 1, 7 and 9 showed continuously rising expression patterns, while Cluster 3, 4 and 8 displayed oppositely declined expression trend successively along with the seed maturation. Among them, Cluster 7 (107) and 9 (282) displayed the most vigorous continuous upward trend, as Cluster 4 (68) was the top continuous downward cluster. In Cluster 5, 138 DEGs tend to increase the transcription specifically in transition from S1 to S2. Compared with S1, the DEGs in Cluster 2 (196) declined the expression only in S3, while DEGs in Cluster 11 (413) seemed to increase the expression more sharply in S3 than in S2.

### 3.4 KEGG and GO analysis of differentially expressed genes

To gain more insight into the biological function of these genes, KEGG and GO analysis were conducted and the functional enrichment results were obtained as shown in Supplementary Figures S3–S7. Among the upward trend clusters, Cluster 7 was significantly associated with ubiquitin mediated proteolysis (3) by KEGG analysis. By GO enrichment, Cluster 7 was more associated with DNA binding (28) and protein heterodimer activity (20) in molecular function, and nucleosome assembly (21) and cell proliferation (6) in biological process. Cluster 9 was dominantly enriched with ribosome (11), starch and sucrose metabolism (8), carbon metabolism (8) and carbon fixation in photosynthetic organisms (7) by KEGG analysis. The GO analysis of Cluster 9 showed enrichment of DNA binding (20), structural constituent of ribosome (10) and protein heterodimer activity (8) in molecular function, and nucleosome assembly (10), regulation of cell cycle (8), and cell proliferation (7) in biological process. The downward trend clusters were particularly enriched into protein processing in endoplasmic reticulum (9) in Cluster 4, diterpenoid biosynthesis (4) and glutathione metabolism (4) in Cluster 3 by KEGG analysis. Yet by GO analysis of Cluster 4, protein folding (8), response to hydrogen peroxide (7), response to high light intensity (7) in biological process were enriched. Cluster 3 was enriched with oxidation-reduction process (16), response to oxidative stress (4) in biological process, and iron ion binding (13), heme binding (10) and transcription factor activity (8) in molecular function. Cluster 5 (138) was particularly enriched with plant hormone transduction (4) and starch and sucrose metabolism (3) by KEGG analysis, and oxidation-reduction process (13) in biological process and copper ion binding (4) in molecular function by GO enrichment. For Cluster 11, ribosome (22), ribosome biosynthesis in eukaryote (12), RNA transport (7) and cysteine and methionine metabolism (6) were particularly associated by KEGG analysis, and translation (23), carbohydrate metabolic process (11) and nucleosome assembly (10) in biological process and DNA binding (31), structural constituent of ribosome (26), protein heterodimer activity (10) and heme binding (9) in molecular function were enriched by GO analysis. Cluster 2 was significantly associated with protein processing in endoplasmic reticulum (9) by KEGG analysis, unfolded protein binding (6) and nutrient reservoir (5) in molecular function, response to stress (10) and protein folding (7) in biological process by GO enrichment. In summary, these results together indicated that remarkable dynamics of chromatin structure, active ribosome biosynthesis and cell proliferation took place along with the seed maturation, which was accompanied with considerable elevated protein synthesis, DNA binding and carbohydrate metabolism. Yet, with the filling process, the seeds went through lower level of protein processing and secondary metabolite synthesis.

### 3.5 Analysis of key genes involved in the seed development of Tartary buckwheat

#### 3.5.1 Dynamic transcriptome analysis of phytohormone signaling pathway genes involved in seed development

**FIGURE 7:**
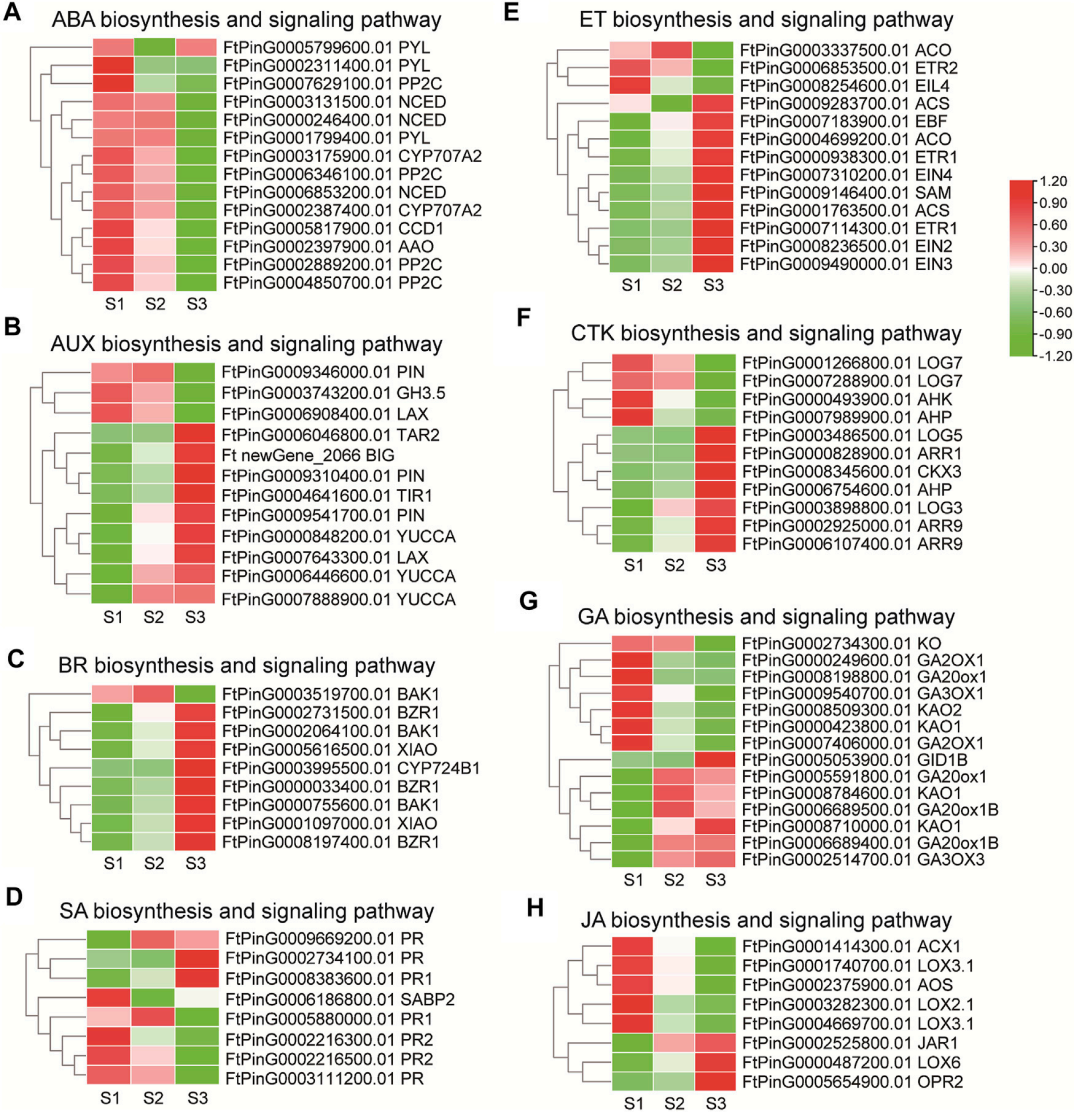
The transcriptome dynamic of genes involved phytohormone biosynthesis and signaling pathways during grain filling in Tartary buckwheat **(A)**, ABA; **(B)**, AUX; **(C)**, BR; **(D)**, SA; **(E)**, ET; **(F)**, CTK; **(G)**, GA; **(H)**, JA.

Phytohormones play notable roles in seed development in rice, maize and Arabidopisis (Santner et al., 2009). Several DEGs were assigned to “plant hormone signal transduction” (ko04075) in the transcriptome data by KEGG enrichment analysis. Genes involved in phytohormone biosynthesis and signaling pathways were thus elaborately analyzed. For ABA biosynthesis (Figure 7A), 3 *NCED*, which encode enzymes catalyzing the rate-limiting step in ABA biosynthesis, and one *AAO*, displayed a descending tendency of expression in maturation phase. Among the ABA signaling genes, 4 *PP2C* gradually down-regulated the expression with the seed growth, while the transcription of 2 *PYL* decreased significantly in S2, and the other *PYL* decreased in S3. Together these results were in support of active ABA signaling in the early seed development. For AUX biosynthesis (Figure 7B), the expression level of one *TAR2* was up-regulated in S3 specifically, with 2 *YUCCA* (*FtPinG0006446600.01*, *FtPinG0007888900.01*) being up-regulated since S2, and one *YUCCA* (*FtPinG0000848200.01*) being up-regulated along the seed growth. Additionally, the AUX catabolic gene, *GH3.5* (*FtPinG0003743200.01*) was down-regulated, which together indicated AUX accumulation in the grain filling seeds. Consistently, one *LAX* (*FtPinG0007643300.01*), 2 *PIN* (*FtPinG0009541700.01*, *FtPinG0009310400.01*) and one *BIG* responsible for AUX transport gradually enhanced the transcription mildly. For the signal transduction, the expression of the AUX receptor-encoding gene *TIR1* (*FtPinG0004641600.01*) significantly increased with the seed growth. Together these results suggested active AUX signaling in the filling process of Tartary buckwheat seeds. Several genes in the ET biosynthesis and signaling displayed differential expression (Figure 7E), as one *SAM*, one *ACO* (*FtPinG0004699200.01*) and 2 *ACS* involved in ET biosynthesis, one *EIN2*, one *EIN3*, one *EIN4*, 2 *ETR1* and one *EBF* involved in ET signaling pathway, strengthened the transcription especially in S3 phase, yet one *ETR2* (*FtPinG0006853500.01*) and one *EIL4* (*FtPinG0008254600.01*) genes involved in signaling transduction decreased the expression significantly with the seed growth. As in Figure 7C, several genes involved in BR biosynthesis and signaling pathways were identified to be differentially regulated. One *CYP724B1* gene (*FtPinG0003995500.01*), homologous to *D11* in rice (Tanabe et al., 2005), involved in BR biosynthesis increased its transcription in maturation phase. 2 DEGs (*FtPinG0005616500.01*, *FtPinG0001097000.01*) homologous to *XIAO* in rice (Jiang et al., 2012) were moderately up-regulated with the seed growth, while 2 *BAK1* (*FtPinG0002064100.01*, *FtPinG0000755600.01*) and 3 *BZR1* raised their expression significantly with seed growth, providing insight of positive roles of BR during seed filling. Genes in the CTK pathways displayed variated expression (Figure 7F). The expression of 2 *LOG* (*FtPinG0003486500.01*, *FtPinG0003898800.01*) involved in biosynthesis, and one *AHP* (*FtPinG0006754600.01*), 3 *ARR* involved in signaling pathway were up-regulated particularly in S3 phase, while 2 *LOG* (*FtPinG0001266800.01*, *FtPinG0007288900.01*), one *AHK* and one *AHP* (*FtPinG0007988900.01*) declined the expression especially in S3 phase indicating regulation by CTK in the early or late filling stages. For genes involved the GA biosynthesis (Figure 7G), one *KO* decreased the expression in maturation phase. 2 *KAO* (*FtPinG0008509300.01*, *FtPinG0000423800.01*), one *GA2OX1* (*FtPinG0008198800.01*), one *GA3OX1* (*FtPinG0009540700.01*) decreased the expression since middle filling stage. Yet the expression of 2 *KAO* (*FtPinG0008710000.01, FtPinG0008784600.01*), 3 *GA20ox1* (*FtPinG0005591800.01*, *FtPinG0006689400.01*, *FtPinG0006689500.01*), and one *GA3OX3* (*FtPinG0002514700.01*) were up-regulated since middle filling phase. The *GID1B* encoding the GA receptor in the signaling transduction was moderately up-regulated in S3 (Figure 7G). These results suggested complicated GA signaling transduction during seed maturation. Among the DEGs involved in JA biosynthesis and signaling (Figure 7H), one *LOX6*, one *OPR2* genes increased the expression significantly, while one *LOX2*, 2 *LOX3*, one *AOS* and one *ACX1* gene declined the expression with the seed maturation. No specific DEG was identified to be involved in JA signaling pathway. One *SABP*2 (*FtPinG0008383600.01*) and 3 *PR* (*FtPinG0002734100.01*, *FtPinG0008383600.01*, *FtPinG0009669200.01*) involved in SA signaling raised the expression with the seed filling (Figure 7D), as the transcription of *PR* (*FtPinG0002216300.01*, *FtPinG0002216500.01*, *FtPinG0003111200.01, FtPinG0005880000.01*) declined with the seed growth. No DEGs involved in SA biosynthesis was discovered. These results together pointed to less participation of JA and SA signaling pathways in seed filling.

#### 3.5.2 Dynamic transcriptome analysis of transcription factors during seed development

**FIGURE 8:**
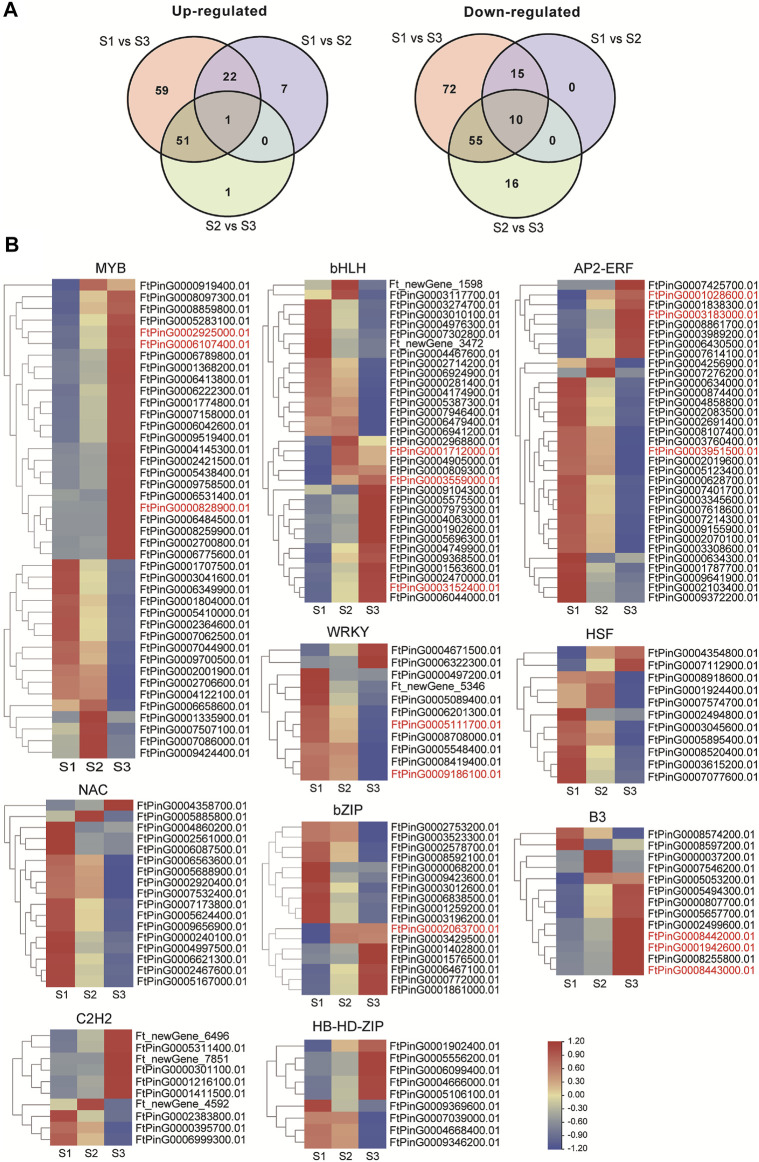
Transcriptome dynamic of transcription factors during seed development. **(A)**. Venn map showing the differentially up-regulated or down-regulated TFs. **(B)**. Heatmap illustration of top 10 family TF DEGs clustered by gene families (MYB; bHLH; AP2/ERF; NAC; bZIP; B3; HSF; WRKY; C2H2; and HD-HB-ZIP). The TFs involved in phytohormone signaling or with high similarity with characterized genes involved in seed size control in other plants were marked in red color.

Transcription factors (TFs) could participate in many aspects of cellular processes in seed development, thus the expression dynamics of total TF genes involved in seed development of Tartary buckwheat were carefully investigated. According to the RNA-seq data, a total of 1077 TFs were identified as expressed in at least one developmental stage. An overview of the transcription factors that were differentially regulated was shown in Supplementary Figure S8. In total, 309 TFs were taken as DEGs, with 7 being identified as new TF genes. Additionally, 87 DEGs encoding *RLK-Pelle* family kinases and 45 DEGs transcription repressors involved in transcription regulation showed remarkable expression variation. Briefly, the top 10 TF families with the largest numbers were MYB (42), bHLH (33), AP2/ERF (33), NAC (17), bZIP (17), B3 (13), HSF (11), WRKY (11), C2H2(10), and HD-HB-ZIP (10) (Supplementary Figure S8). Both up- and down-regulation of these TF genes occur in the process of seed maturation. Compared with S1, 23 TF genes were commonly up-regulated in S2 and S3, with 7 TF genes being up-regulated specifically to S2, and 59 TF DEGs being up-regulated specifically to S3 (Figure 8A). Additionally, compared with S1, 25 TF genes were commonly down-regulated in S2 and S3, with 72 TF genes being down-regulated specifically to S3. The expression of the top 10 family TF DEGs was displayed in Figure 8B. 9 out of 11 DEGs in WRKY and NAC families were down-regulated with seed growth, with the other 2 being up-regulated in S2 or S3 phase, which indicated WRKY and NAC DEGs may function mainly in the early stage of seed development or negatively during grain filling. Special attention was paid to the TFs involved in phytohormone signaling or with high similarity with characterized genes involved in seed size control in other plants (marked in red color). The 3 *ARF* (*FtPinG0008443000.01*, *FtPinG0001942600.01*, *FtPinG0008442000.01*) of B3 family and 4 *DOF* (*FtPinG0000702500.01*, *FtPinG0008252900.01*, *FtPinG0001221400.01*, *FtPinG0006052400.01*) of C2C2 family involved in AUX signaling pathway increased the expression either in middle filling stage or the maturation stage. Moreover, the *ABI5* (*FtPinG0002063700.01*) of bZIP family in ABA signaling and the *ARR* (*FtPinG0000828900.01*, *FtPinG0002925000.01*, *FtPin G0006107400.01*) of MYB family involved in CTK signaling displayed ascending transcription pattern with the seed growth. The *ERF* (*FtPinG0003951500.01*, *FtPinG0003183000.01*, *FtPin G0001028600.01*) of AP2/ERF family, homologous to *ANT* (Meng et al., 2015) and *AP2* (Jofuku et al., 2005) in rice, and the *bHLH* (*FtPinG0003559000.01*, *FtPinG0003152400.01*, *FtPinG0001712000.01*), homologous to *Awn-1* (Luo et al., 2013) in Arabidopsis showed remarkable variation in S3 phase in Tartary buckwheat seeds, whereas the *WRKY* (*FtPinG0009186100.01*, *FtPinG0005111700.01*), homologous to *WRKY53* (Tian et al., 2017) involved in seed size were mainly down-regulated with the seed growth.

#### 3.5.3 Dynamic transcriptome analysis of expansin family proteins during seed development

**FIGURE 9:**
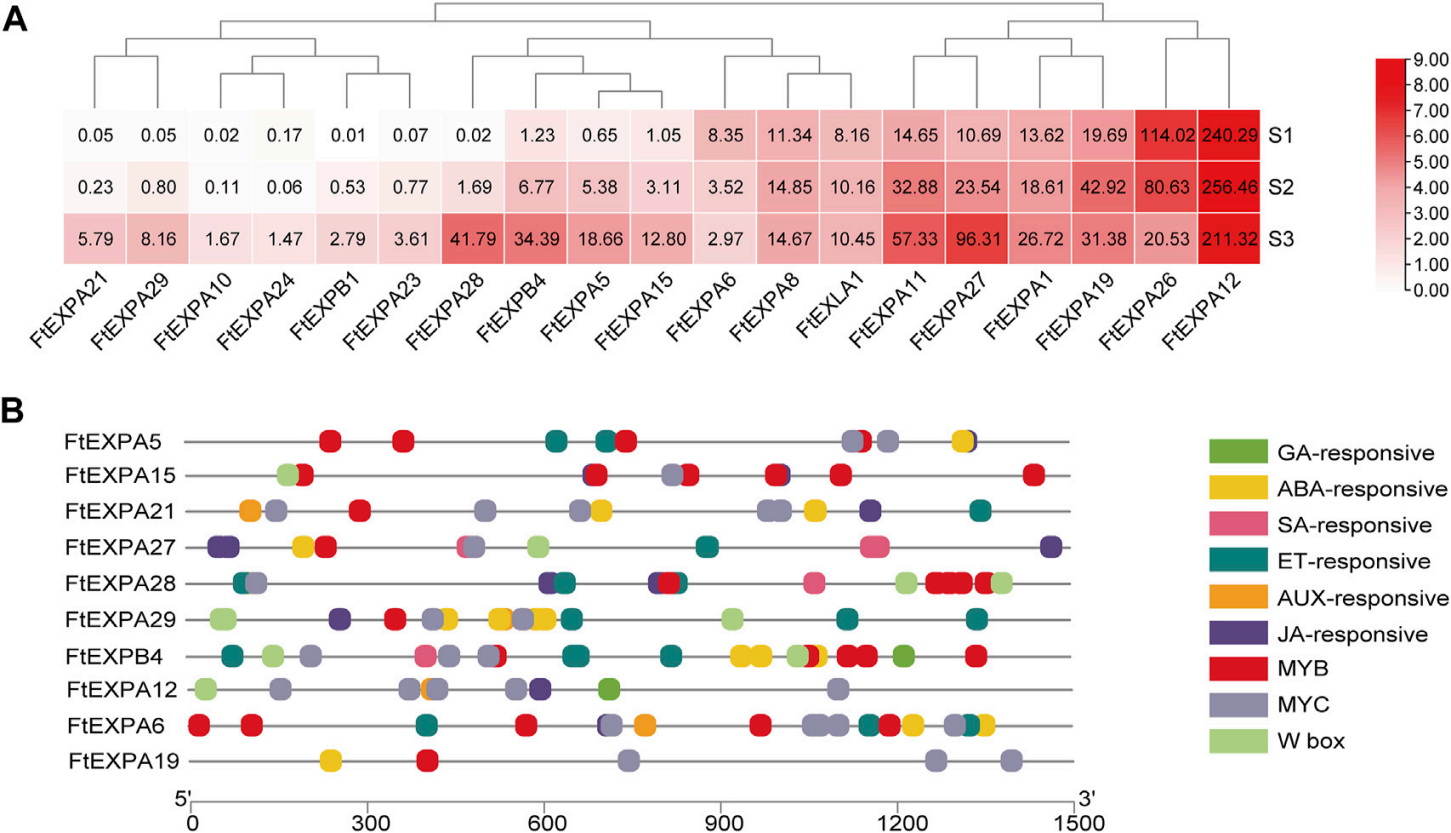
Transcriptome dynamics of expansin family proteins in the seed development. **(A)**. Heatmap representation of the transcriptomic dynamic of differentially expressed expansin genes during seed maturation. The values in the heatmap indicate the FPKM value of the DEGs. **(B)**. Illustration of seed development associated *cis*-acting elements in the promoter region of differentially expressed expansin genes.

**FIGURE 10:**
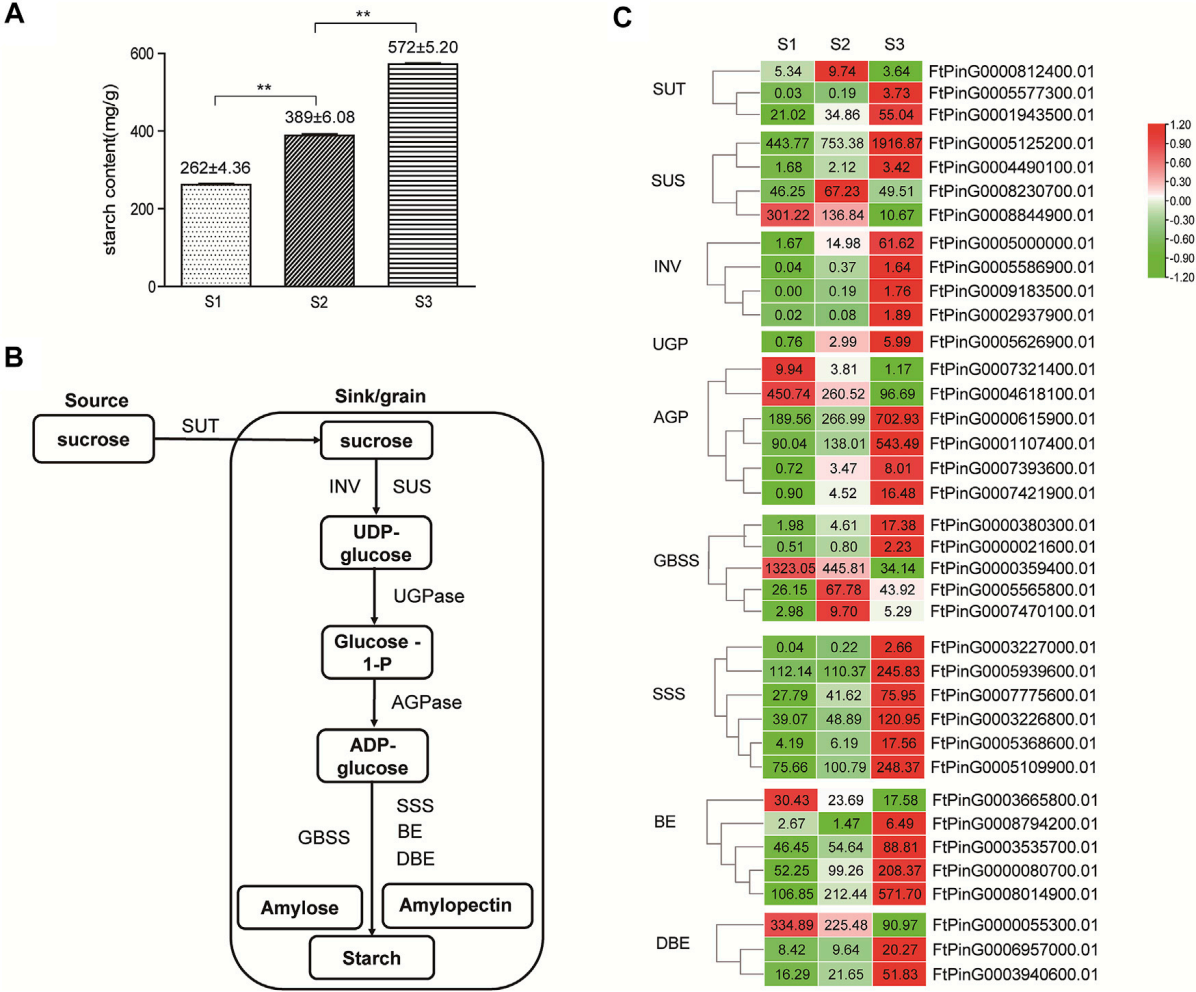
Starch synthesis in Tartary buckwheat seeds. **(A)**. The amount of total starch in Tartary buckwheat seeds. **(B)**. Schematic representation of starch biosynthesis in the seeds. **(C)**. Expression pattern of starch biosynthesis genes that were differentially expressed during seed development (The genes with FPKM≥1 in at least one stage are shown, and the original FPKM value were marked in the heatmap).

Seed size changed distinctly with the development of growth stages in Tartary buckwheat seeds (Figure 1B), with the highest length and width at maturation phase. Plant expansin genes belong to a group of loosening proteins located in the cell wall, and was an important component for cell expansion (Cosgrove, 2015). Previously, expansin was reported to function fundamentally in seed size and yield determination in many plants (Cosgrove, 2015). To explore the roles of expansin family genes in Tartary buckwheat, the transcription dynamics during the grain filling stage were mined here. As in Figure 9A, out of the genome wide 37 expansin genes, 19 were expressed in seeds (with FPKM>1). Among them, the most abundant gene in dehulling seeds was *FtEXPA12*. *FtEXPA12*, *FtEXPA8* and *FtEXLA1* were constantly expressed. As 14 expansin genes substantially displayed an increased trend of expression along with the seed growth, only 2 genes (*FtEXPA6* and *FtEXPA26*) declined their expression notably. Genes with variated expression were analyzed subsequently. As in Figure 10A, among the 16 differentially expressed genes, 14 were classified into subfamily-A, with 2 being classified into subfamily-B (*FtEXPB4*, *FtEXPB1*). Further correlation analysis showed that most of the up-regulated expansin genes were strongly correlated with each other (Supplementary Figure S9). Among the up-regulated expansin genes, the transcription level of *FtEXPA5*, *FtEXPA11*, *FtEXPA15*, *FtEXPA21*, *FtEXPA27*, *FtEXPA28*, *FtEXPA29* and *FtEXPB4* reached the top in seed maturation stage, while *FtEXPA19* was transcribed most in middle filling stage and slightly decreased in seed maturation stage. Nevertheless, *FtEXPA5*, *FtEXPA15*, *FtEXPA21*, *FtEXPA27*, *FtEXPA28*, *FtEXPA29* and *FtEXPB4* displayed the most significant transcriptional variations along with the seed growth (Figure 9A). Interestingly, these genes were phylogenetically quite close, as *FtEXPA5, FtEXPA28*, *FtEXPA29* and *FtEXPA27* were classified in the same branch in the previous report (Sun et al., 2021).

Downstream effecting genes involved in seed development were usually regulated by upstream phytohormone signals and transcription regulators. To explore the transcriptional regulation of expansin genes in Tartary buckwheat seeds, the upstream 1.5 Kb promoter sequences of expansin genes were subsequently subjected to Plantcare for *cis*-regulatory element analysis. As shown in Figure 9B, aside from the consensus eukaryotic promoter elements, such as the TATA-box and CAAT-box, light-responsive elements and stress-responsive elements (*cis*-acting regulatory element essential for the anaerobic induction, MBS in drought-inducibility, and WUN-motif involved in wound-responsiveness) were also discovered in the promoters. To be noted, *cis*-regulatory elements, such as ERE, ABRE, TGACG-motif, CGTCA-motif, P-box and TCA-element responsive to phytohormone ET, ABA, JA, SA, GA, and AUX were notably over-represented in the promoter region of expansin DEGs. Transcription binding sites of MYB, MYC and WRKY TFs were also frequently dispersed. Among the most transcribed genes, the significant differentially expressed genes *FtEXPA5*, *FtEXPA15*, *FtEXPA27*, *FtEXPA28* and *FtEXPB4* were analyzed. Particularly, the promoter region of *FtEXPA27* was richer in SA- and JA-responsive elements, while the *FtEXPA28* promoter bared more ET- and JA-responsive elements and MYB and WRKY binding sites. In the *FtEXPA5* promoter, 4 MYB and 2 MYC binding sites and 2 ET-responsive elements were found, while 6 MYB binding sites and 2 JA-responsive elements were discovered in the *FtEXPA15* promoter region. The B-type expansin *FtEXPB4* promoter seemed to have relatively more TF binding sites than A-type expansin genes and was quite rich in ET- and ABA-responsive elements, and MYB, MYC and WRKY binding sites, with an additional GA-responsive *cis*-acting element. *FtEXPA12* had 5 MYC binding sites and one GA-responsive element in the promoter. MYC is important component of the JA signaling pathway. Thus, the above data provided clues that phytohormone ABA, ET, JA, GA and SA, and TF MYB, MYC, and WRKY probably participated in expansin gene regulation during seed development in Tartary buckwheat.

To further explore the relationship of phytohormone with expansin genes in the process of seed maturation, the DEGs in the ABA, ET, GA, AUX, JA and SA phytohormone signaling pathways were then subjected to correlation analysis with the variated expansin genes. From our research, BR was found to promote Tartary buckwheat grain filling rate (Wei, 2021), thus DEGs in BR biosynthesis and signaling pathway were also included for correlation analysis. As shown in Supplementary Figure S10, most up-regulated expansin genes, except *FtEXPA19*, showed strong positive correlations with DEGs in ET (Supplementary Figure S10A), BR (Supplementary Figure S10E) and AUX (Supplementary Figure S10F) signaling pathways. Yet the declined expansin gene *FtEXPA26* was in strong negative correlation with most differentiated genes in ET (Supplementary Figure S10A), BR (Supplementary Figure S10E) and AUX (Supplementary Figure S10F) signaling pathways. Moreover, most up-regulated expansin genes, except *FtEXPA19*, displayed strong negative correlations with DEGs in ABA signaling pathways, while *FtEXPA26* was in strong positive correlation (Supplementary Figure S10B). Nevertheless, strong positive or negative correlations were only found between up-regulated expansin genes (except *FtEXPA19*) and 2 *PR* in the SA signaling pathway, and *OPR2* (*FtPinG0005654900.01*) and *LOX6* (*FtPinG0000487200.01*) in JA signaling pathway, and *GID1B* (*FtPinG0005053900.01*) and *KAO1* (*FtPinG0008710000.01*) in the GA signaling pathway. Together, these results preferred regulation of expansin family genes by phytohormone ET, ABA, AUX and BR more potentially than GA, JA and SA, in the process of grain filling. Additionally, the above TFs that were homologs of genes involved in seed size determination in other plants (Figure 9, red marked TFs) were subjected to correlation analysis with variated expansin genes. As in Supplementary Figures S11, 3 *ARF* and 2 *DOF* (*FtPinG0000702500.01*, *FtPinG0008252900.01*) involved in AUX signaling pathway, 2 *ARR* in CTK signaling pathway, one *bHLH* (*FtPinG0003152400.01*) homologous to *Awn-1* in Arabidopsis and 2 *ERF* (*FtPinG0003183000.01*, *FtPinG0001028600.01*) homologous to *ANT* and *AP2* in rice displayed strong correlation with differentially expressed expansin genes, except *FtEXPA19*, *FtEXPA6* and *FtEXPA26.* Yet, the *Awn-1* homologous *bHLH* (*FtPinG0001712000.01*) correlated strongly with *FtEXPA19*. These results suggested possible positive transcriptional regulation of *expansin* by these TFs during Tartary buckwheat grain filling. Nevertheless, the 2 *WRKY*, homologous to *WRKY53*, and the *ERF* (*FtPinG0003951500.01*) involved in seed size represented strong negative correlation with most up-regulated *expansin*, indicating negative regulatory roles.

#### 3.5.4 Analysis of starch biosynthesis gene expression in seed development

With the remarkable change of seed shape along with the development, both the fresh and dry weight of grains companionly elevated significantly (*p* < 0.05) (Figure 1C), reaching the top at stage 3. Starch was the major form of carbohydrates accumulated at mature seeds and the main nutrient that make the seeds and other storage organs expand and enlarge (Saripalli and Gupta, 2015). Therefore, the amount of starch largely determined the final grain yield in plants. The level of total starch in the grain filling stage seeds of Tartary buckwheat was subsequently measured. As shown in Figure 10A, the starch amount indeed increased remarkably along with the process of seed maturation from 262 ± 4.36 mg/g to 572 ± 5.20 mg/g, in accordance with severe change of seeds both in width and dry weight (Figure 1). According to above KEGG analysis, “Starch and sucrose metabolism” were dominantly enriched in significantly variated co-expression clusters (Cluster 2, 5, 9) (Supplementary Figures S4, S5, S7). So, genes involved in starch and sucrose metabolism were proposed to significantly affect the grain filling process and final yield in Tartary buckwheat. Thus, the transcriptional profile of genes involved in starch and sucrose metabolism were thoroughly explored. Among them, 61 genes were significantly differentially expressed, with 34 belonging to glycosyl hydrolases family (Supplementary Figure S12). Among the 7 DEGs encoding glucan endo-1,3-β-glucosidase, the expression of *FtPinG0000387400.01* increased only in middle filling stage and *FtPinG0002200200.01* specifically decreased in late filling stage, as the expression of the other 5 DEGs gradually increased with the seed growth. 10 β-glucosidase-encoding genes were identified, with 5 being continuously up-regulated with grain filling, 2 being significantly down-regulated in S3 phase, and 3 being down-regulated with the seed growth. Both 2 DEGs encoding α-glucosidase increased their expression in S3 phase. Notably, 2 out of the 4 genes encoding pectinesterase identified were remarkable down-regulated in S3. 3 DEGs encoding amylase displayed notable decline along with the seed filling, indicating low level of starch hydrolysis. Moreover, 4 genes encoding trehalose-phosphate phosphatase displayed significant transcriptional dynamic. Several genes involved in starch biosynthesis were identified noticeably and were discussed below.

In plants, sucrose was transported from source, such as leaves, to the sink for the synthesis of starch by sucrose transport protein (SUT) (Figure 10B). In our transcriptome data, 3 *SUT* displayed differential expression pattern (Figure 10C), with *FtPinG0001943500.01* being mostly transcribed and continuously up-regulated moderately with seed growth. Starch biosynthesis in seeds initiated with the transition of sucrose to UDG-glucose by sucrose synthase (SUS) and invertase (INV). As in Figure 10C, among the 4 differentially expressed sucrose synthase genes, *FtPinG0005125200.01* represented the highest expression level and continuously upward trend along with the seed maturation, as *FtPinG0008230700.01* were up-regulated in S2 phase only and *FtPinG0008844900.01* was down-regulated with the seed growth. 4 *INV* increased the expression significantly, as *FtPinG0005000000.01* and *FtPinG0008078700.01* were insoluble cell wall invertases, and the other 2 were soluble vacuolar invertases. Among them, *FtPinG0005000000.01* displayed the highest expression level and an increased pattern with the seed maturation. Only one *UGP* (*FtPinG0005626900.01*) was differentially expressed in seeds and showed an up-regulated tendency with the grain filling. The formation of ADP-glucose from glucose-1-phosphate by AGP, is considered as the committed rate-limiting step of starch biosynthesis (Saripalli and Gupta, 2015). 6 *AGP* displayed differential expression along with the seed growth, with 4 being gradually up-regulated and 2 being gradually down-regulated. Among them, *FtPinG0000615900.01* and *FtPinG0001107400.01* were transcribed at the highest level in mature seeds, as *FtPinG00004618100.01* displayed the highest expression in the early phase. 5 *GBSS* were differentially expressed in filling stage seeds, with 2 (*FtPinG0005565800.01*, *FtPinG0007470100.01*) being transcribed most at middle filling stage, and 2 (*FtPinG0000380300.01*, *FtPinG0000021600.01*) gradually increased the expression until the seed maturation. The *GBSS* (*FtPinG0000359400.01*) was the exception, which showed the highest expression out of the 5 in early filling stage. 6 *SSS* displayed differentially expression, and all reached the top expression level at maturation phase. 4 out 5 differentiated *BE* genes increased their expression with the seed growth, yet *FtPinG0000080700.01* and *FtPinG0008014900.01* were most transcribed in mature seeds. *FtPinG0006957000.01* and *FtPinG0003940600.01* in the *DBE* DEGs reached the highest expression levels in maturation phase. These above results indicated that up-regulated genes involved in starch biosynthesis may participate in the starch accumulation process during Tartary buckwheat grain filling, while down-regulated genes may function in the early stage of seed development.

To clarify the regulation of structural genes involved in starch biosynthesis, *cis*-regulatory element analysis of the 1.5 Kb promoter region was also carried out by Plantcare. As shown in Supplementary Figures S13, S14, similar with expansin genes, aside from the consensus eukaryotic promoter elements, such as the TATA-box and CAAT-box, light-responsive elements, and stress-responsive elements (*cis*-acting regulatory element essential for the anaerobic induction, MBS in drought-inducibility, and WUN-motif involved in wound-responsiveness), hormone-responsive *cis*-regulatory elements, such as ERE, ABRE, TGACG-motif, CGTCA-motif and TCA-element responsive to phytohormone ET, ABA, JA, SA, GA, and AUX, and transcription binding sites of MYB, MYC and WRKY TFs were also over-represented. These results indicated the candidate regulation of starch biosynthesis by these phytohormone and TFs.

#### 3.5.5 Verification of RNA-Seq gene expression by RT-PCR

**FIGURE 11:**
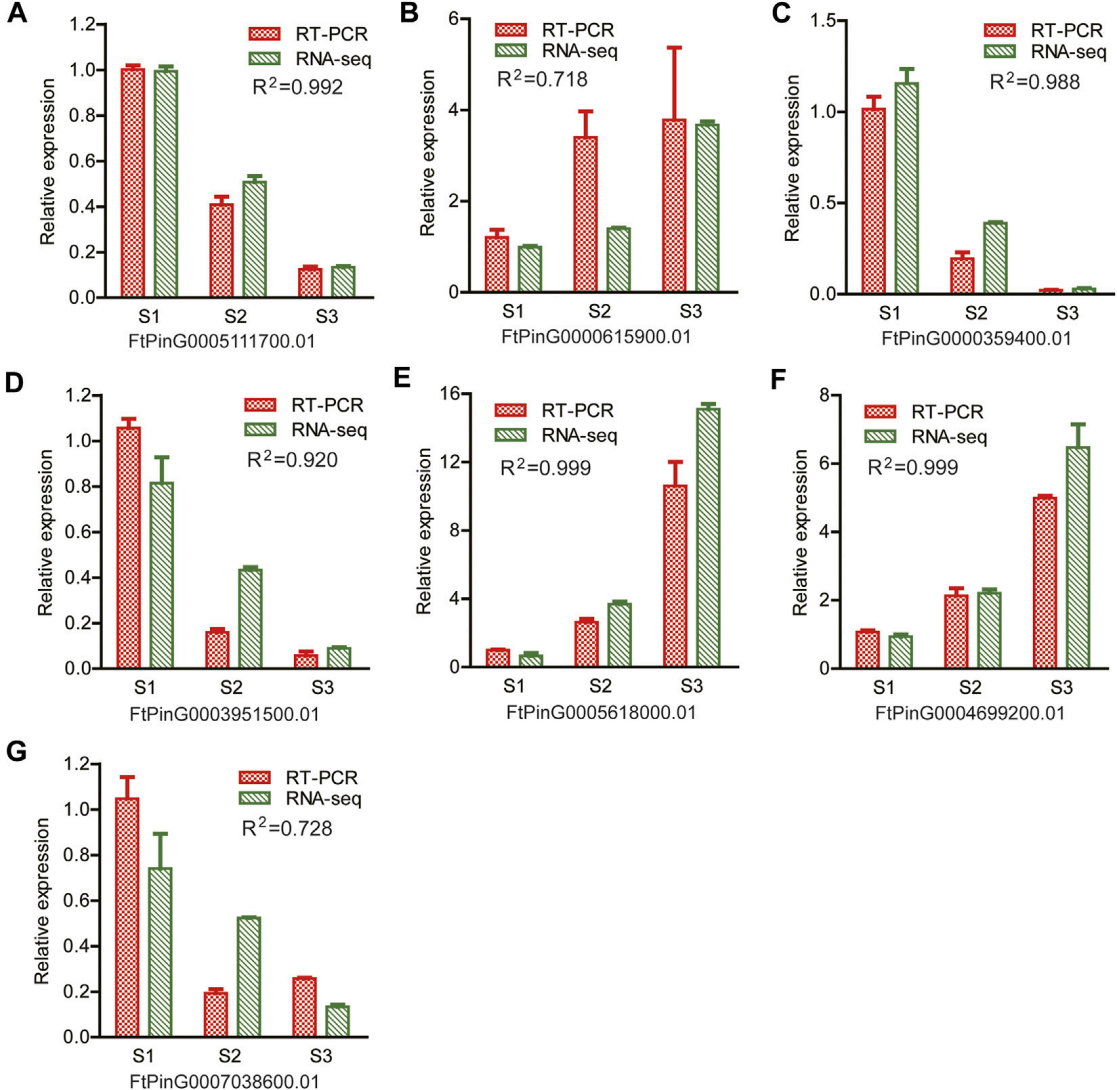
Illustration of the relative expression of 7 randomly selected genes for RT-PCR verification. R^2^ value indicated the correlation coefficient of the relative expression levels between the RT-PCR results and the RNA-seq by Pearson correlation analysis **(A)** FtPinG0005111700.01; **(B)** FtPinG0000615900.01; **(C)** FtPinG0000359400.01; **(D)** FtPinG0003951500.01; **(E)** FtPinG0005618000.01; **(F)** FtPinG0004699200.01; **(G)** FtPinG0007038600.01.

A quantitative RT-PCR was performed to verify the reliability of the RNA-seq data. Seven DEGs were randomly selected, as displayed in Figure 11. Among them, *ACO* (*FtPinG0004699200.01*), *AGP* (*FtPinG0000615900.01*), *ERF* (*FtPinG0003951500.01*) and *WRKY* (*FtPinG0005111700.01*) were included as representatives. The expression of these DEGs were consistent with the results of the RNA-seq data (R^2^ > 0.7), confirming the reproducibility of the transcriptome data.

## 4 Discussion

Various nutritional and pharmacological effects of Tartary buckwheat have been extensively studied (Li and Zhang, 2001; Huda et al., 2021; Zhang et al., 2021). Nevertheless, poor post-embryonic grain filling in Tartary buckwheat largely halts the grain yield improvement. Few reports were about the gene regulatory network governing the physiological changes during the filling stage of Tartary buckwheat seeds. Thus, in this study we tried to sort out the key seed development genes based on our transcriptome data from different developmental stages of the dehulling filling stage seeds of Tartary buckwheat (*F. tataricum* cv. Xiqiao No.1). Sharp transitions in gene expression occurred along with the grain filling (Figures 2–4), consistent with significant physiological changes with seed growth in Tartary buckwheat (Figure 1). 4249 DEGs (with FC > 4) were identified totally that may be key genes related to seed development.

Phytohormones play indispensable roles in seed development in rice, maize and Arabidopisis (Santner et al., 2009). AUX and ABA function notably in controlling the embryogenesis pattern and promoting the accumulation of storage products during the subsequent filling stage (Schussler et al., 1984; Moller and Weijers, 2009; Chandrasekaran et al., 2014). Liu et al. (2018a) found the ascending tendency of ABA in the process of Tartary buckwheat fruit maturation and the variation of GA and AUX. Here, significant variation of genes in the signal transduction pathways of phytohormones was observed (Figure 7). Yet genes involved in ABA biosynthesis and signaling displayed a descending pattern of expression, indicating active ABA signaling in the early seed development (Figure 7A). Increase of AUX biosynthesis genes (*TAR* and *YUCCA*) and decrease of catabolic genes (*GH3.5*) (Figure 7A) suggested AUX accumulation during grain filling. The AUX transport genes (*LAX*, *PIN* and *BIG*) strengthened the expression in Tartary buckwheat kernel. These results strongly implied positive regulatory roles of AUX along with grain filling process in Tartary buckwheat. As the balance of AUX and ABA was proposed to be key factors regulating the cell division rate in the early seed development of buckwheat (Liu et al., 2018b), and evidence indicated synergistic regulation of cell expansion *via* both AUX and GAs (Fenn and Giovannoni, 2021), the nexus of ABA, AUX, GA and other hormones during grain filling required further elucidation. Yet variated expression of genes in the GA and CTK biosynthesis and signalling pathways suggested complicated roles by these phytohormones in seed filling process. Overexpression of OsBZR1, a BR-signaling TF, resulted in higher grain yield in rice (Zhu et al., 2015). Here, the BR biosynthesis related *CYP724B1*, *BZR1* and coreceptor BAK1 homologs were up-regulated gradually with the seed growth. As we previously found, appropriate spraying of BR on the leaves of Tartary buckwheat could significantly improve the seed setting rate, grain filling rate, and yield, and reduce the abortion rate of grains notably (Wei, 2021). Thus, it is speculated that BR signaling play positive roles in grain filling. Ethylene can promote fruit ripening, and ethylene signaling plays an important role in fruit development (Persak and Pitzschke, 2014; Fenn and Giovannoni, 2021). Here, genes involved in ET biosynthesis (*SAM*, *ACO*, *ACS*), and signaling (*EIN2*, *EIN3, EIN4*, *ETR1*, *EBF*) enhanced the expression with the seed growth (Figure 7E), indicating consistent positive function of ethylene in filling stage Tartary buckwheat.

Transcriptional regulators were important factors controlling seed size in plants, including TF, transcriptional coactivators, and regulators involved in chromatin modification. The significant enrichment of up-regulated genes in DNA binding, nucleosome assembly and cell proliferation in Cluster 7 and 9 by GO analysis indicated remarkable change of chromatin structure and dynamics along with the seed maturation in Tartary buckwheat, in which transcriptional regulators could be involved. In the maturation process of Tartary buckwheat seeds, 309 TF DEGs were identified with MYB, bHLH, AP2/ERF, NAC, bZIP, B3, HSF, WRKY, C2H2, and HD-HB-ZIP as the top 10 families. MYB, NAC, WRKY, bHLH, MADS and AP2/ERF TFs were reported to regulate of fruit development/maturation (Jakoby et al., 2002; Persak and Pitzschke, 2014; Zinsmeister et al., 2016; Zhang et al., 2018; Liu et al., 2019a; Liu et al., 2019b; Liu et al., 2019c; Ma et al., 2019; Liu et al., 2020). In Tartary buckwheat, several MYB (FtMYB6, FtMYB116, FtMYB3, et al.) were reported to be positively or negatively involved in flavonoid biosynthesis (Yao et al., 2020; Wang et al., 2022), yet no specific TF gene has been characterized to participate in grain filling process directly. Since CTKs are the key drivers of seed yield (Jameson and Song, 2016), the up-regulated *ARR*s involved in CTK signaling could be candidate MYB genes involved in grain filling in Tartary buckwheat. Similarly, with the importance of AUX and ABA in seed maturation in Tartary buckwheat (Liu et al., 2018a), the *ARF* and *DOF*, *ABI5* DEGs involved in AUX and ABA signaling pathways could be targets to improve grain filling. In wheat developing endosperm, NAC019-A1 was a negative regulator of starch synthesis (Liu et al., 2020). Therefore, the decreased NAC DEGs (Figure 8B) were worth for further characterization of whether regulating starch biosynthesis or not. In addition, distinct variation of TF genes homologous to *ANT* and *AP2* in rice, and *Awn-1* in Arabidopsis controlling seed size (Figure 8) were supposed to regulate seed development process either.

Cell walls provide essential plasticity for plant cell division and defense, which are often conferred by the expansin superfamily with cell wall-loosening functions. Recently, expansin family genes were reported to participate in many aspects of plant growth and development processes, such as root hair growth, germination, leaf growth, and grain yield in different plants (Cosgrove, 2015; Calderini et al., 2021). Cotton plants overexpressing GhRDL1 and GhEXPA1 proteins produced strikingly more fruits, larger seed size and doubled seed mass (Xu et al., 2013). Transgenic over-expression of sweet potato expansin gene (*IbEXP1*) in Arabidopsis produced larger seeds, accumulated more protein and starch in each seed, and produced more inflorescence stems and siliques than control plants (Bae et al., 2014; Chen et al., 2016). Targeted expression of *TaExpA6* in the young seed lead to a significant increase in grain size without a negative effect on grain number, and a final yield boost by 10% in wheat under field conditions (Calderini et al., 2021), which provided an opportunity to overcome a common bottleneck to yield improvement across many crops (Cosgrove, 2021). During grain filling of Tartary buckwheat seeds, significant transcriptional variation of expansin genes were discovered during grain filling. Strong correlation of the up-regulated expansin genes indicated the possible common regulation. Phytohormone exerts variated effects on *EXP* expression. Repressive effect by AUX on the expression of *FaEXP2* and *FaEXP5* in Chilean strawberry fruit (Figueroa et al., 2009) and by GA on *CDK-Exp3* transcription in persimmon, and positive regulation of *MiExpA1* expression by ET within a short time in mango (Sane et al., 2005), during fruit softening were reported. GA mediated expansion of floral organs *via* expansins prior to anthesis (Azeez et al., 2010). Transgenic overexpressed wheat *TaEXPB23* in tobacoo, involved in the abiotic stress response, were upregulated by exogenous JA and salt stress, but downregulated by exogenous GA, ET, IAA and alpha-naphthlcetic acid (NAA) (Han et al., 2012). A regulatory module controlling GA-mediated endosperm cell expansion involving spatiotemporal control of the cell expansion gene *AtEXPA2* is critical for seed germination in Arabidopsis (Sanchez-Montesino et al., 2019). In this study, phytohormone (ABA, ET, AUX, JA, SA and GA) responsive elements and MYB, MYC and WRKY binding sites were found in the promoters of the remarkable increased expansin gene. Strong correlation of these expansin DEGs with BR, JA, AUX and ET signaling (Supplementary Figure S10) and the AUX and CTK related TFs (ARF, DOF, ARR) or the seed size related TFs (bHLH, ERF and WRKY) (Supplementary Figure S11) was discovered. Combining these results, we proposed that phytohormone AUX, ABA, ET and BR and their responsive TFs were candidate important regulators of grain filling through targeting expansin genes in Tartary buckwheat. The functions of significantly variated expansin genes were worth for further study which could be candidate targets to improve crop yield in Tartary buckwheat.

As shown in Figure 1, the length and width of seed coat reached a maximum size in S3 phase, which sets an upper limit to final size of a grain (Figure 1A). However, the size of the decorticated seeds continuously expanded until the maturation phase in Tartary buckwheat (Figure 1A, lower picture). As the primary nutrient of buckwheat fruit, starch accounts for 70% of the total substance content (De Bock et al., 2021). Thus, the amount of starch in the filling seeds largely determined the final yield. Consequently, genes involved in starch and sucrose metabolism were proposed to noticeably affect the grain filling process and final yield in Tartary buckwheat. Down-regulation of amylase genes during seed filling depicted low starch hydrolysis, and up-regulation of *SUT* either in the middle filling seeds or the early maturation seeds, supported efficient sucrose translocation from the source to the sink (seed) for starch synthesis during grain filling in Tartary buckwheat (Figure 10). Indeed, remarkable starch accumulation was found during seed development (Figure 10A). In rice and Arabidopisis, genes in the starch biosynthesis pathway have been reported (Saripalli and Gupta, 2015). However, the buckwheat starch biosynthesis genes remained largely uncharacterized, except for *GBSS* (Wang et al., 2014). Through mining the transcriptome data (Cao et al., 2022), 37 candidate genes covering all steps of starch biosynthesis displayed differential expression (Figure 10C). Up-regulation of biosynthetic genes was found in each step with seed maturation. The abundantly transcribed and enhanced genes in the filling stage, such as *FtPinG0005125200.01* of *SUS*, *FtPinG0005000000.01* of *INV*, *FtPinG0000615900.01* and *FtPinG0001107400.01* of *AGP*, *FtPinG0005565800.01*, *FtPinG0007470100.01* of *GBSS, FtPinG0005109900.01*, *FtPinG0005939600.01* and *FtPinG0003226800.01* of *SSS*, and *FtPinG0000080700.01* and *FtPinG0008014900.01* of *BE*, are worth for further study to elucidate the precise chemical mechanisms of these enzymes in starch synthesis in Tartary buckwheat. In view of the increased trend of starch biosynthesis genes until the maturation phase, it is speculated that without the size limit by the seed coat, starch biosynthesis would possibly continue to expand the seed kernel and raise the final grain yield. Thus, procedures to improve the seed coat size, such as by ectopic expression of specific expansin genes (Cosgrove, 2021), may be effective breeding strategy in Tartary buckwheat.

## Data Availability

The datasets presented in this study can be found in online repositories. The names of the repository/repositories and accession number(s) can be found below: https://www.ncbi.nlm.nih.gov/bioproject/PRJNA857365/.
