# Supplementary material for: Dynamic transcriptome analysis suggests the key genes regulating seed development and filling in Tartary buckwheat (Fagopyrum tataricum Garetn.)
Source: Front Genet. 2022 Aug 22;13:990412. doi: 10.3389/fgene.2022.990412 (PMC9441574; doi:10.3389/fgene.2022.990412)
Supplement: Supplementary file 1 [file DataSheet1.docx]

Supplementary Material

# Supplementary Data


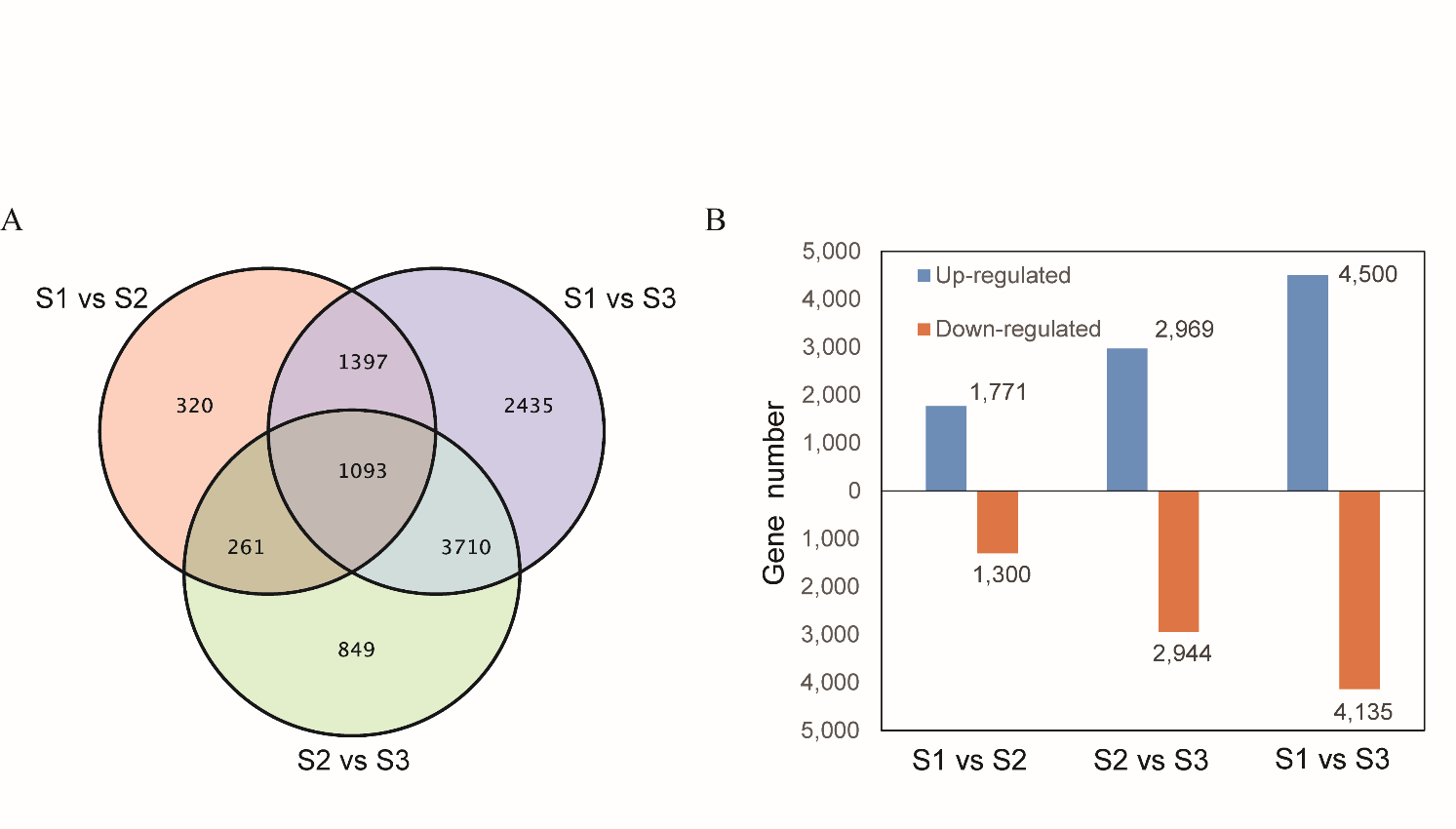


**Supplementary Figure 1.** Venn diagrams showing significantly differentially expressed genes (*p* < 0.01, FC > 2) in comparison groups (**A**). Histogram displaying the up-regulated and down-regulated DEGs (*p* < 0.01, FC > 2) in comparison groups (**B**).


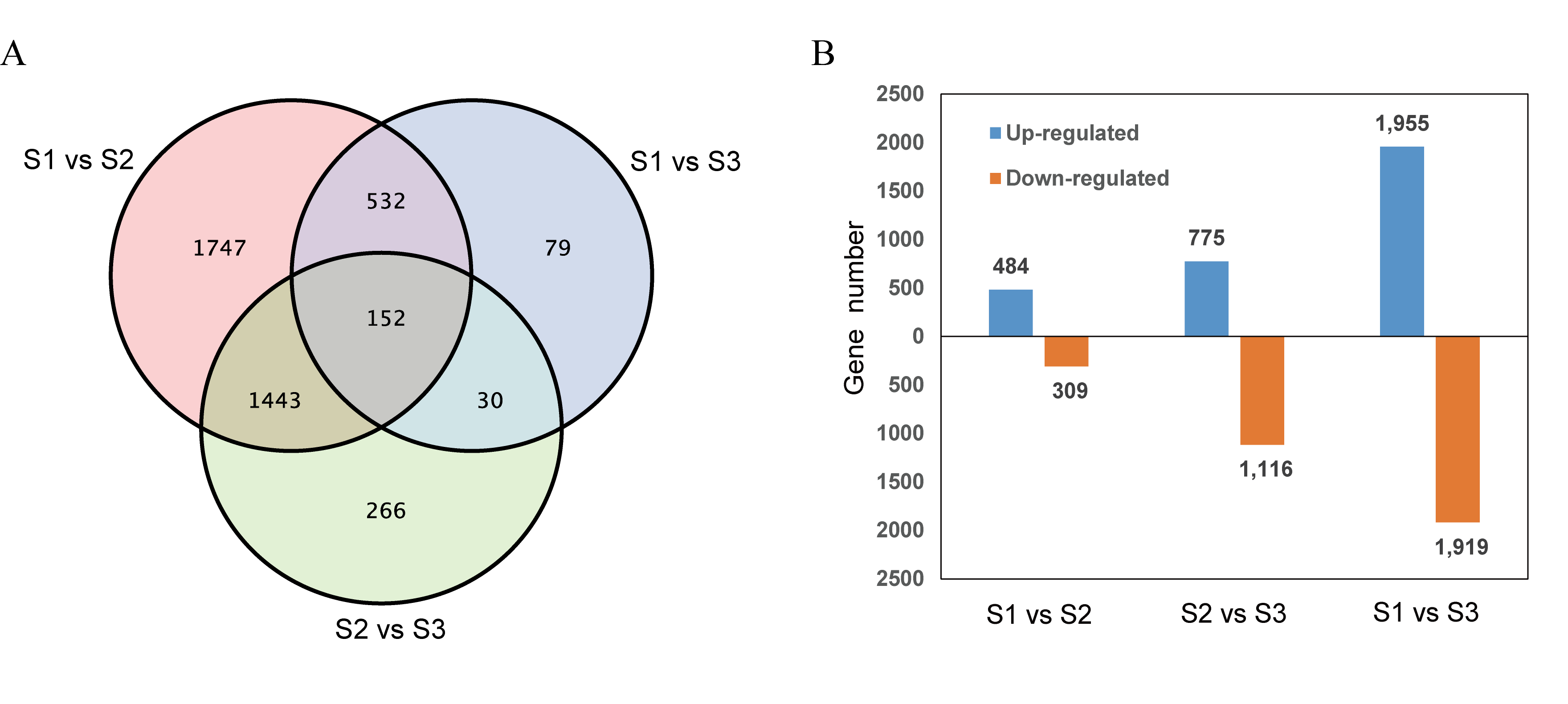


**Supplementary Figure 2.** Venn diagrams showing significantly differentially expressed genes (*p* <0.01, FC > 4) in comparison groups (**A**). Histogram displaying the up-regulated and down-regulated DEGs (*p* <0.01, FC > 4) in comparison groups (**B**).


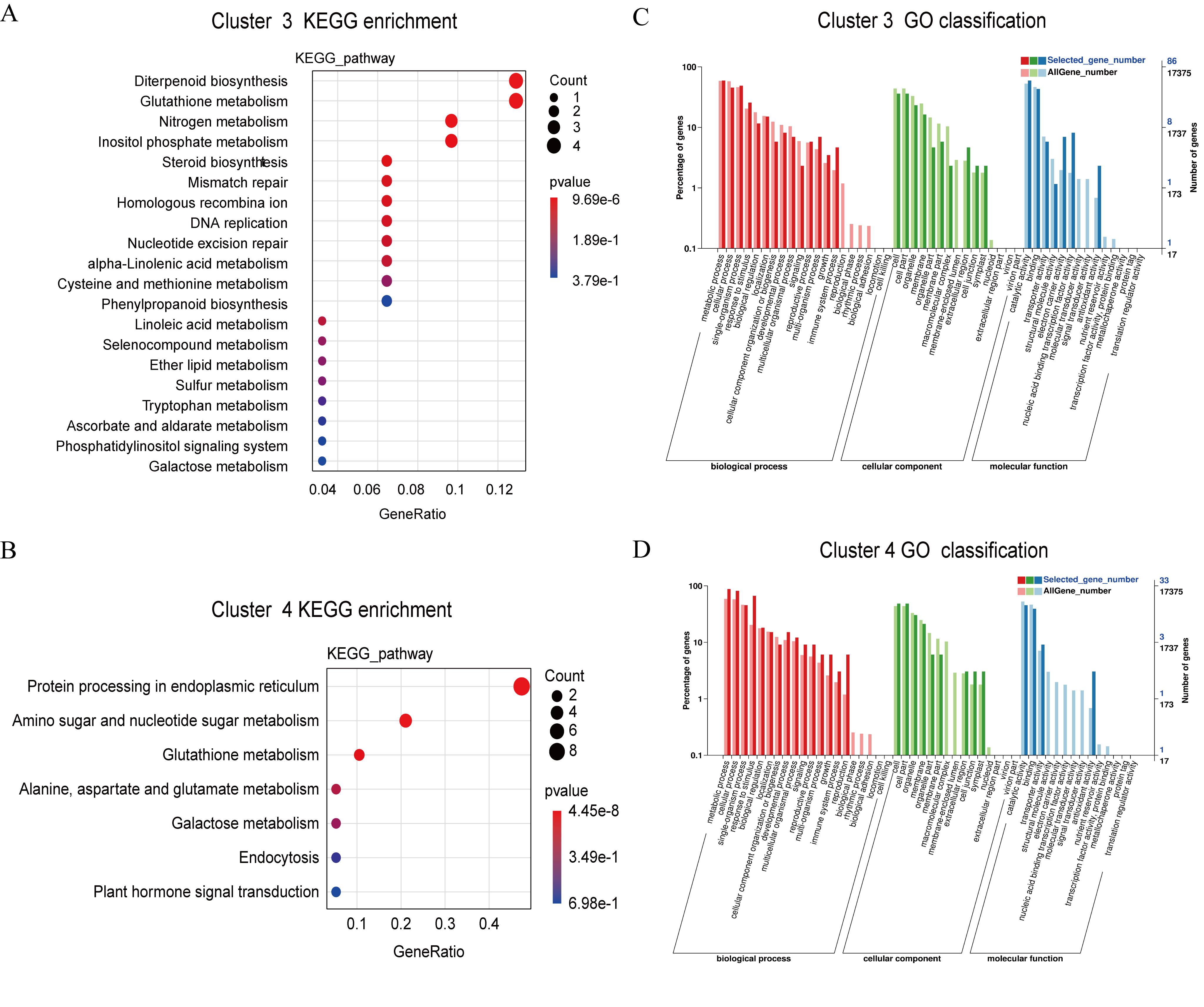


**Supplementary Figure 3.** KEGG and GO analysis of continuously down-regulated clusters in pair-wised comparisons. KEGG enrichment (**A**) and GO classification (**C**) of Cluster 3. KEGG enrichment (**B**) and GO classification (**D**) of Cluster 4.


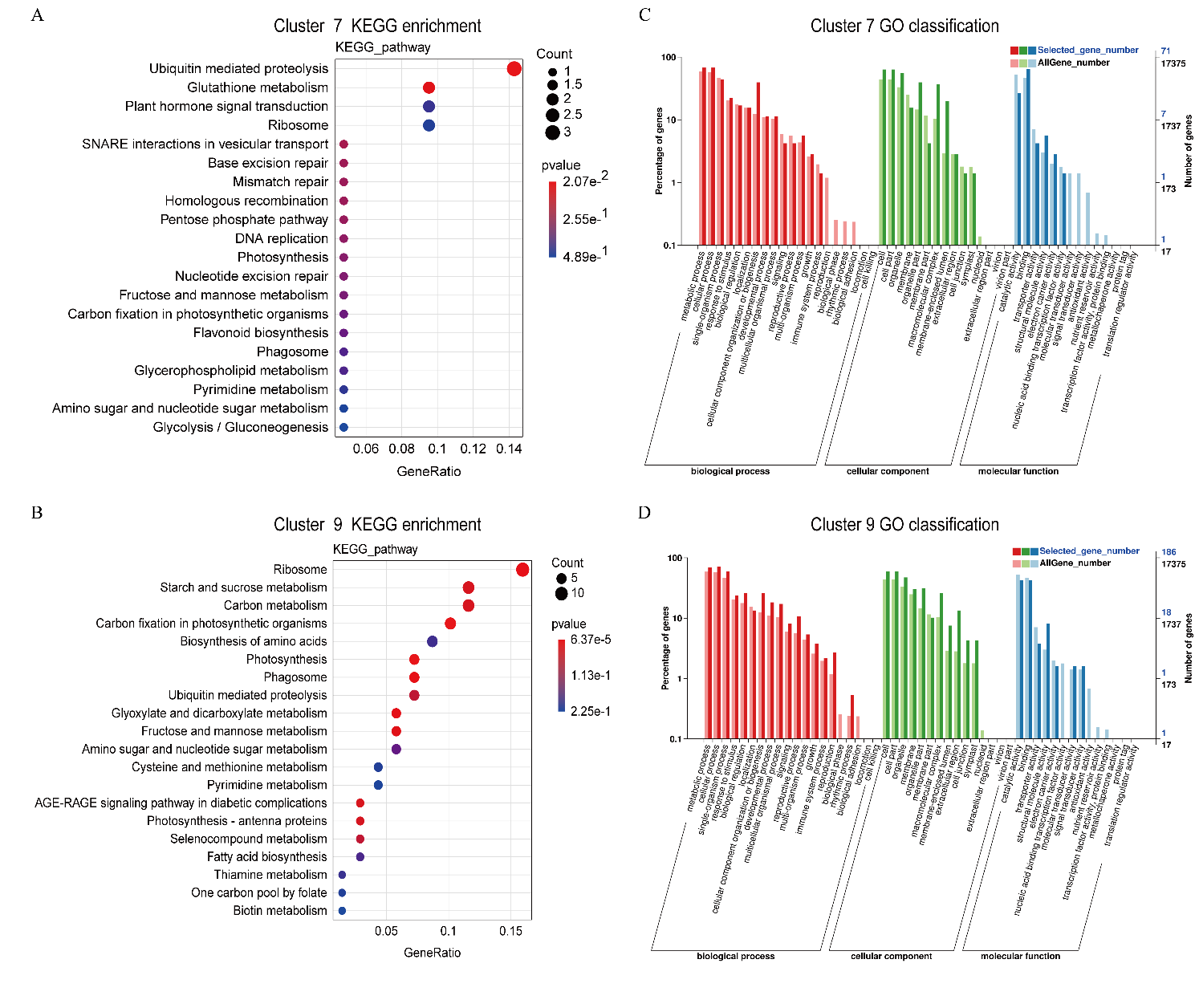


**Supplementary Figure 4.** KEGG and GO analysis of continuously up-regulated clusters in pair-wised comparisons. KEGG enrichment (**A**) and GO classification (**C**) of Cluster 7. KEGG enrichment (**B**) and GO classification (**D**) of Cluster 9.


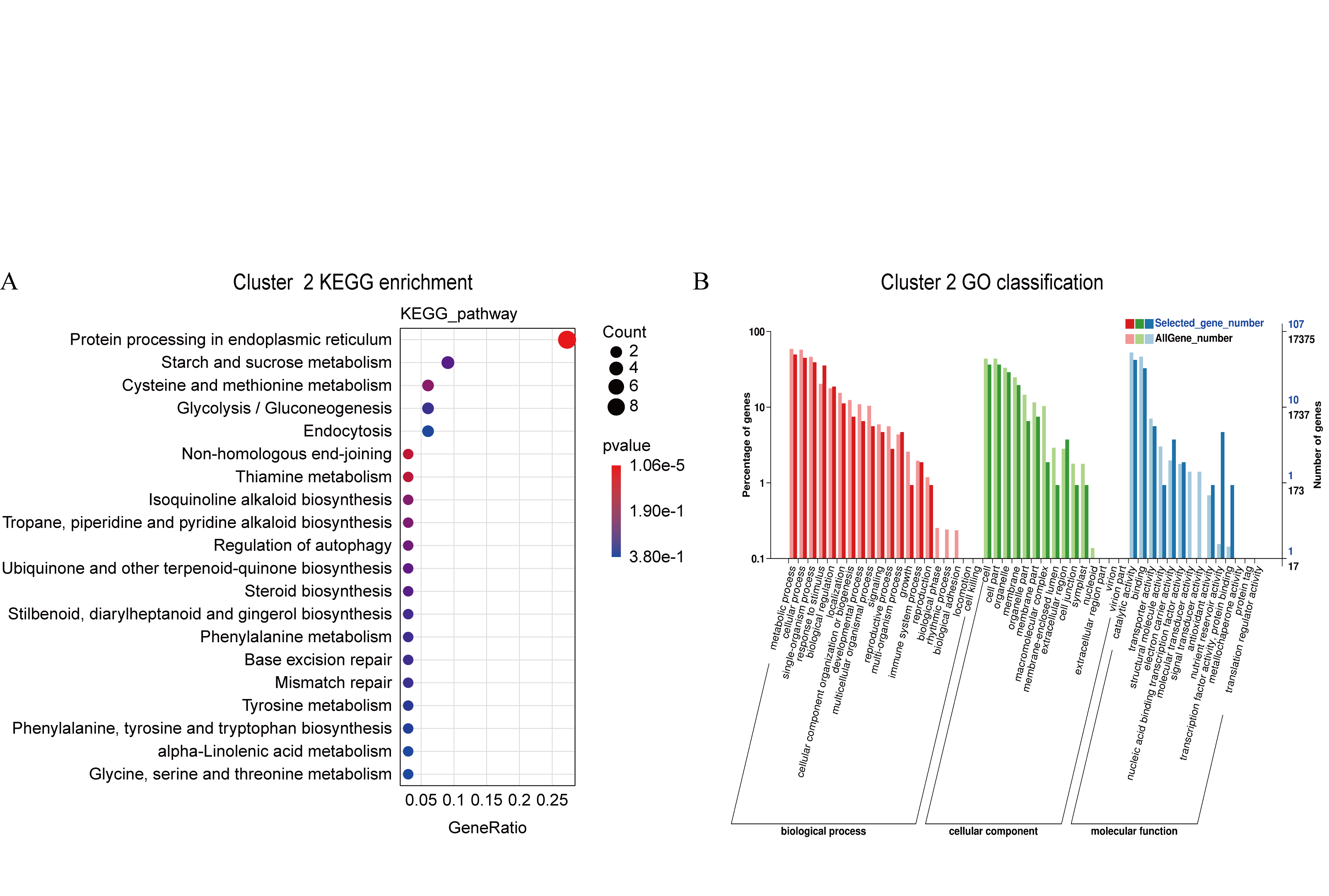


**Supplementary Figure 5.** KEGG and GO analysis of DEGs in Cluster 2. KEGG enrichment (**A**) and GO classification (**B**) of Cluster 2.


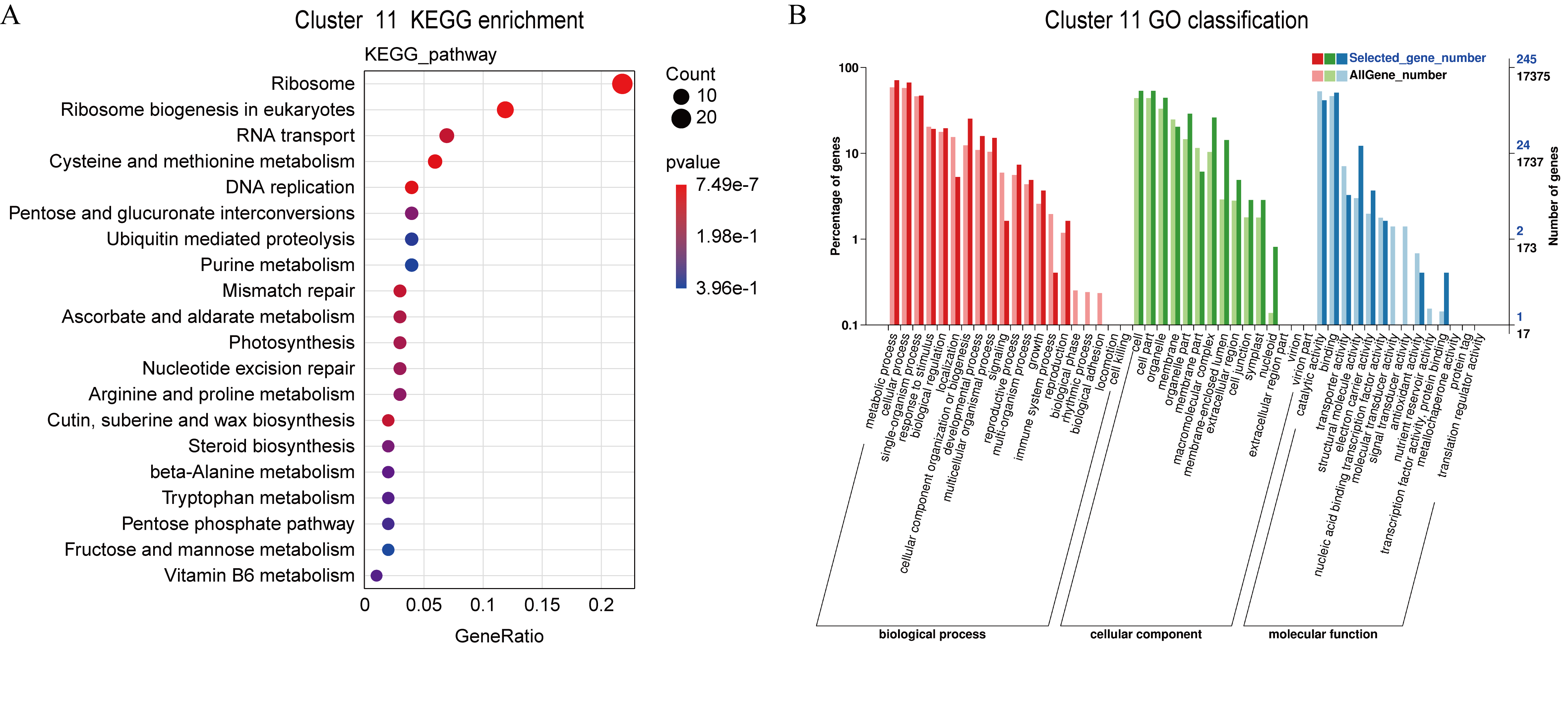


**Supplementary Figure 6.** KEGG and GO analysis of DEGs in Cluster 11. KEGG enrichment (**A**) and GO classification (**B**) of Cluster 11.


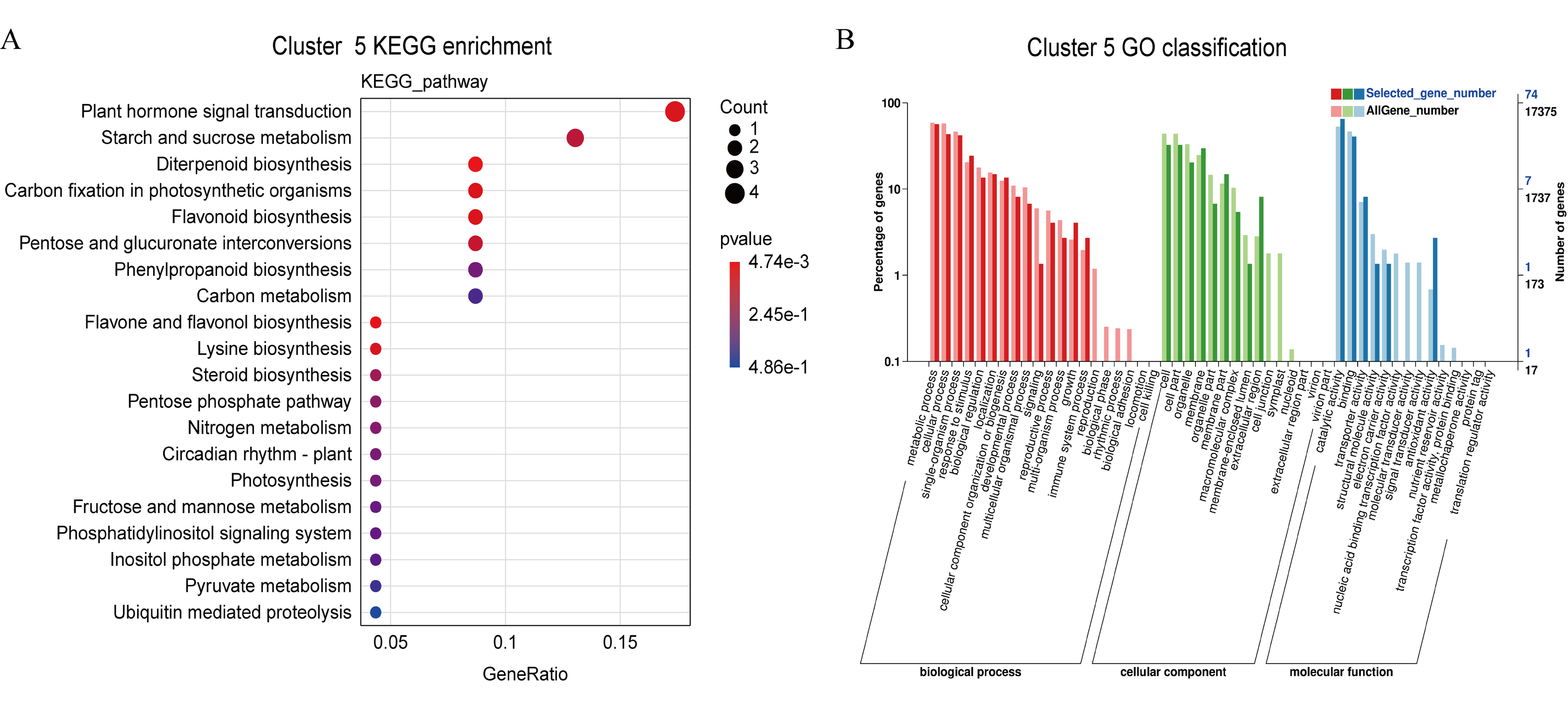


**Supplementary Figure 7.** KEGG and GO analysis of DEGs in Cluster 5. KEGG enrichment (**A**) and GO classification (**B**) of Cluster 5.


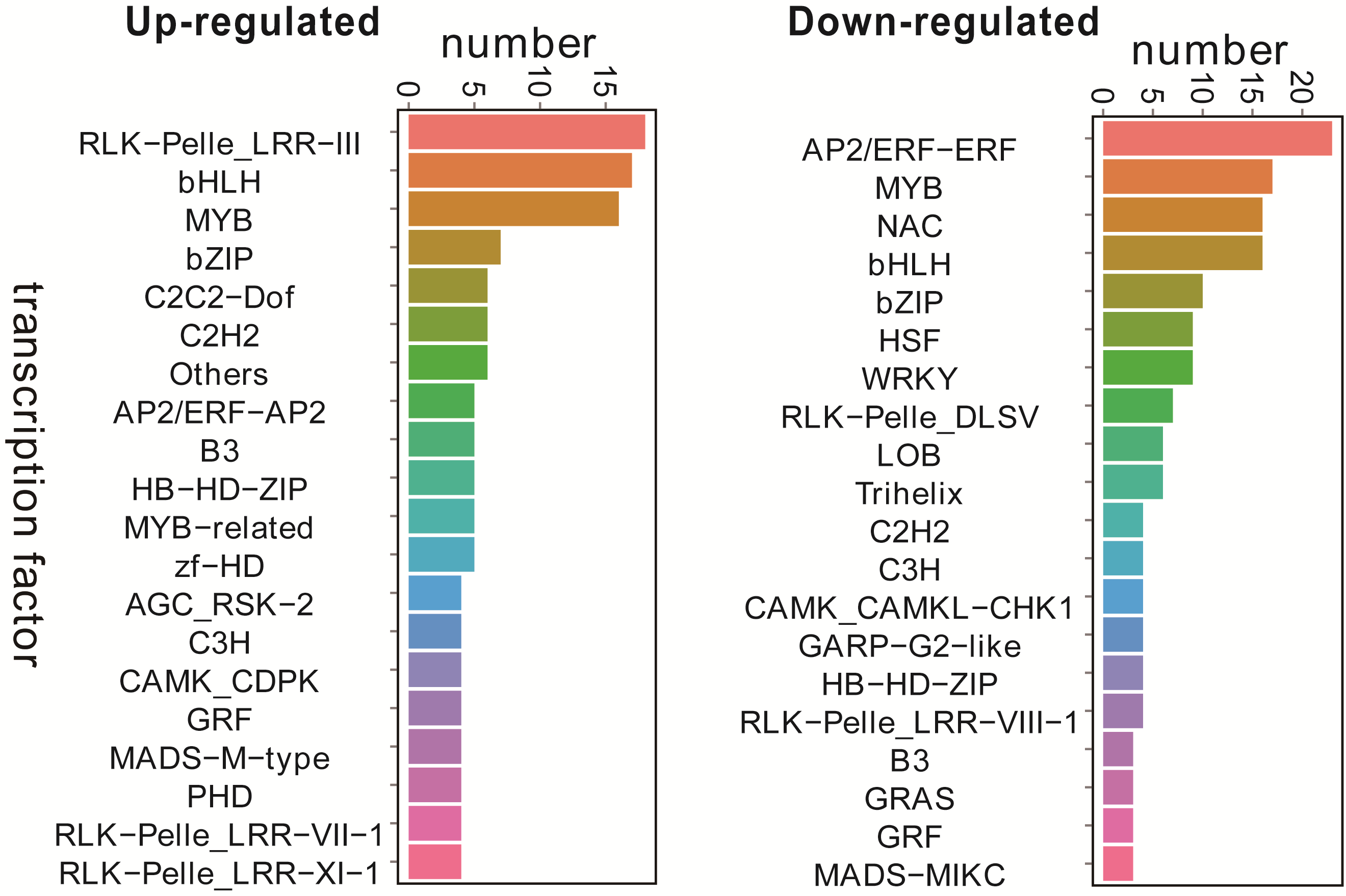


**Supplementary Figure 8.** Histograms displaying the number of the up-regulated and down-regulated TF DEGs classified by families in comparison groups.


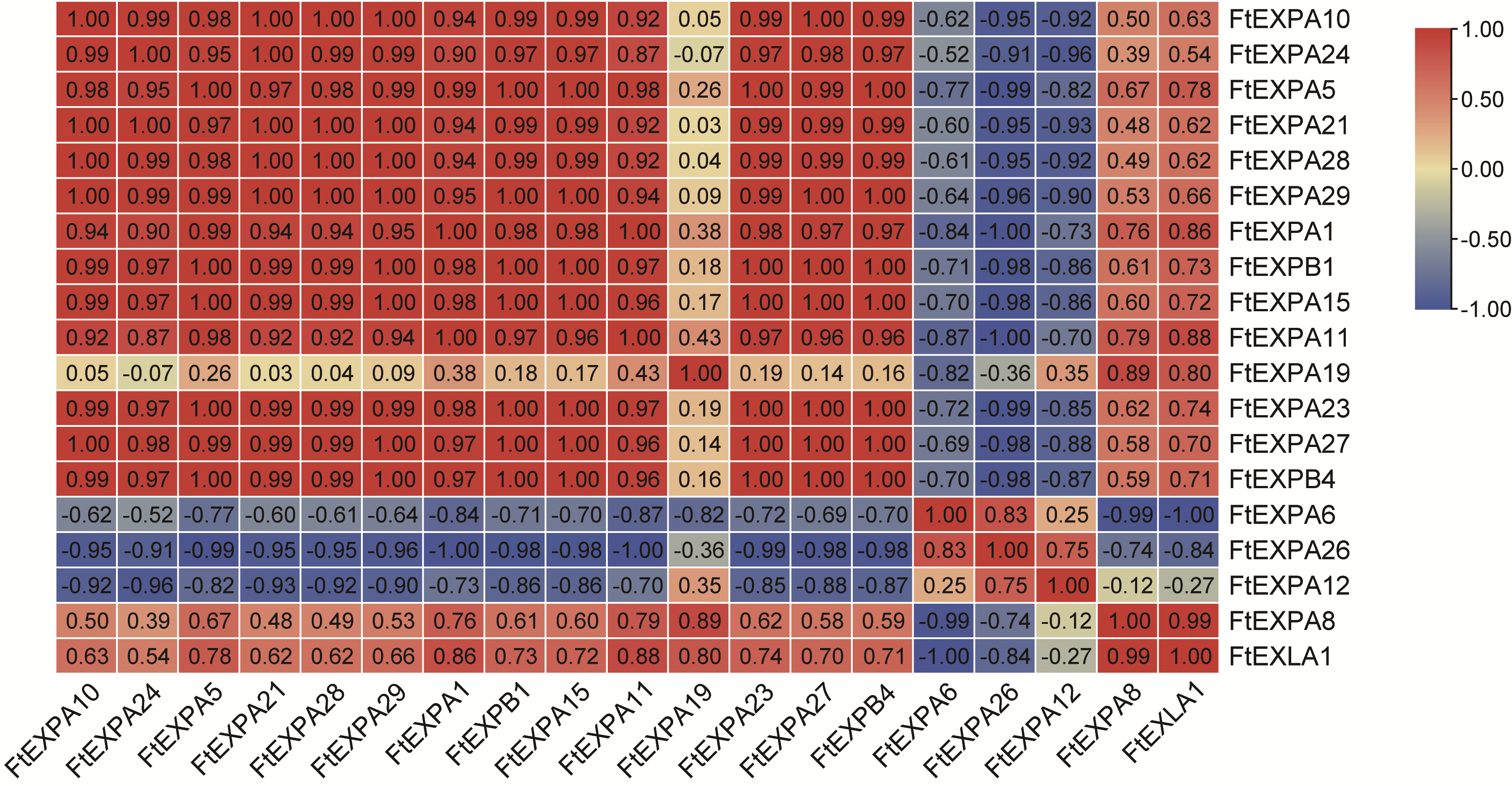


**Supplementary Figure 9.** The correlation between differentially expressed expansin genes. The values in the heatmap indicate the correlation coefficient by the Pearson correlation analysis.


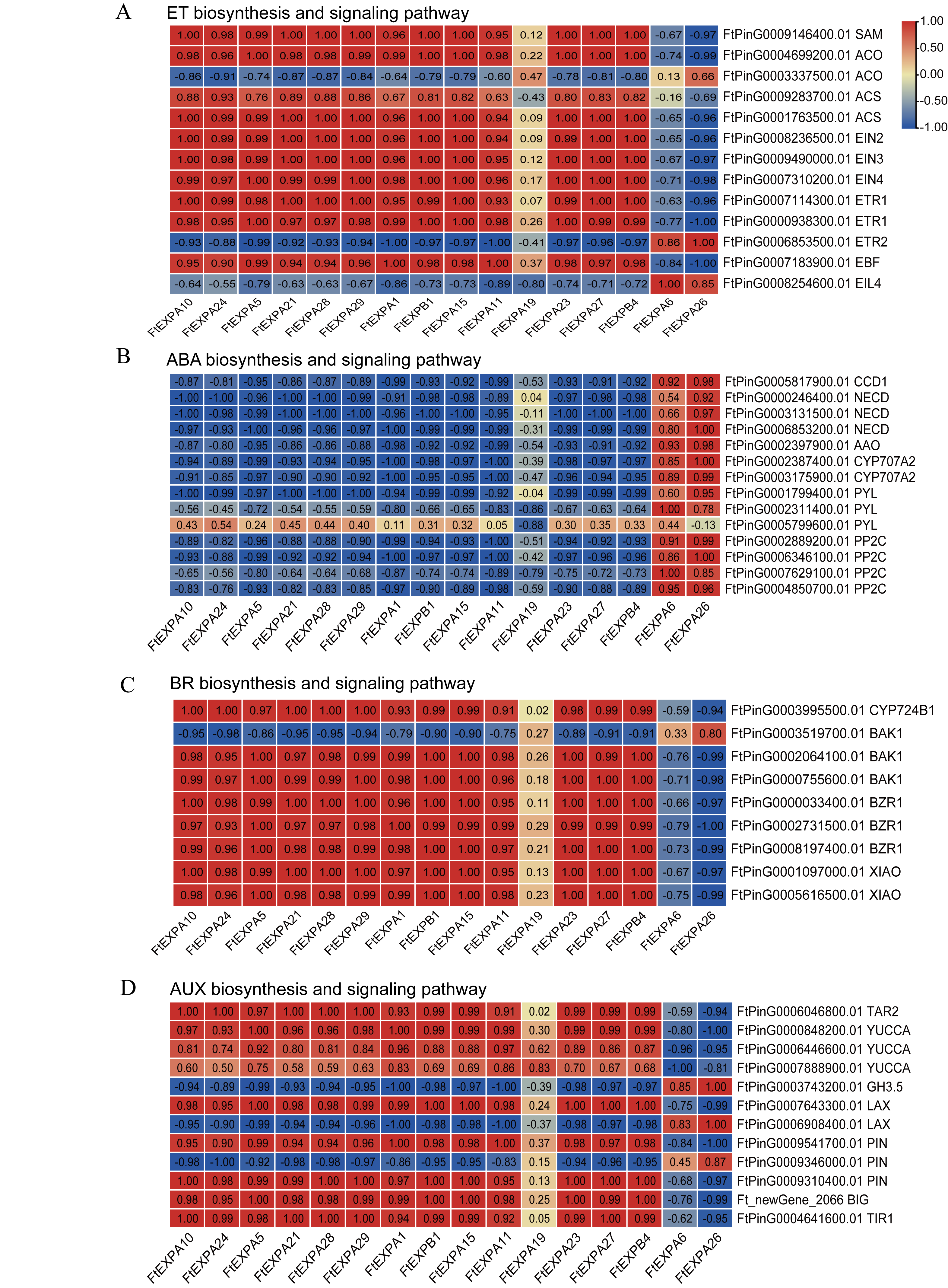


**Supplementary Figure 10.** Correlation analysis of differentially expressed expansin genes with DEGs involved in phytohormone biosynthesis and signaling pathway (**A**. ET; **B**. ABA; **C**. BR; **D**. AUX). The values in the heatmap indicate the correlation coefficient by the Pearson correlation analysis.


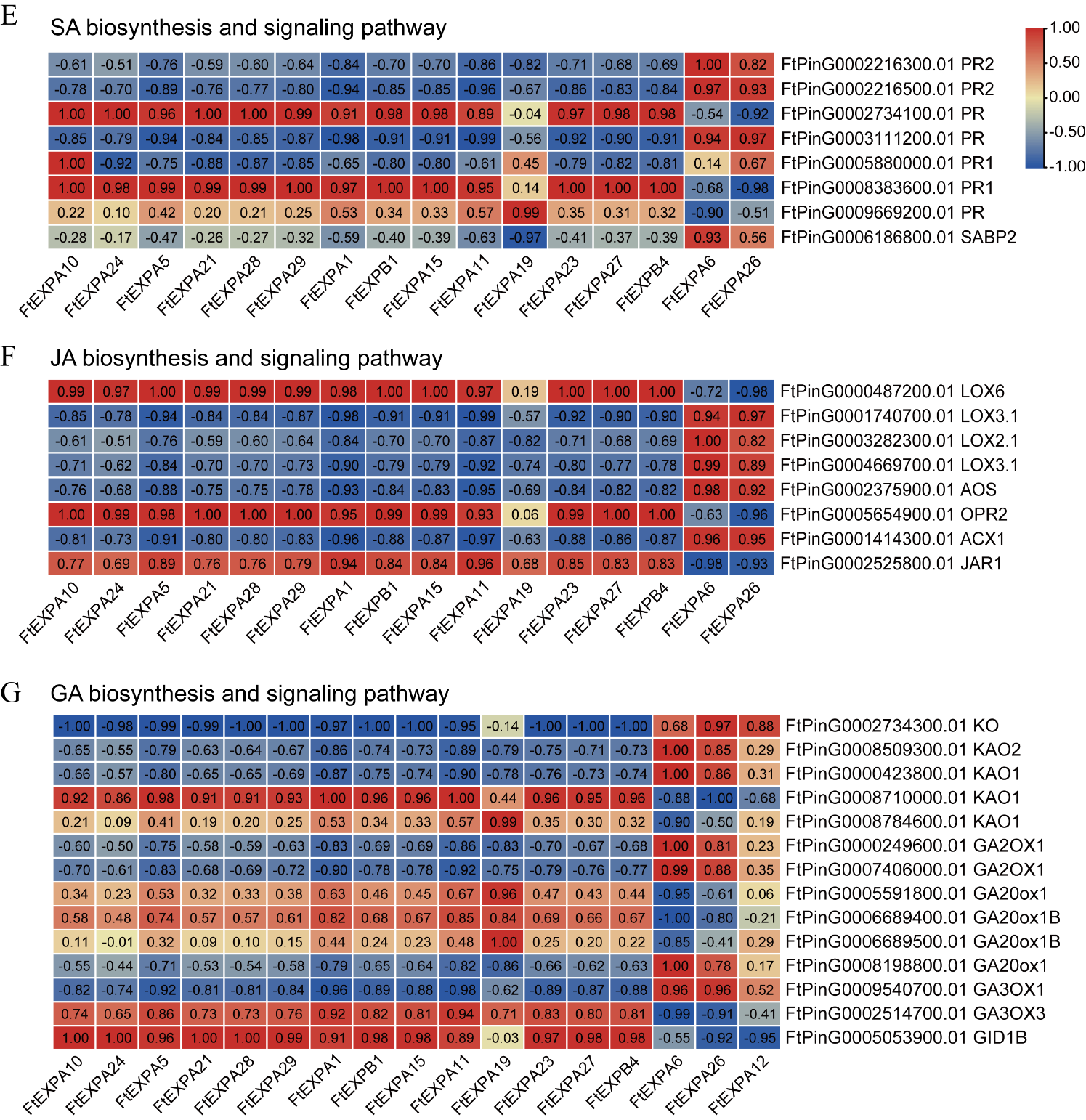


**Supplementary Figure 10 continued.** Correlation analysis of differentially expressed expansin genes with DEGs involved in phytohormone biosynthesis and signaling pathway (**E**. SA; **F**. JA; **G**. GA). The values in the heatmap indicate the correlation coefficient by the Pearson correlation analysis.


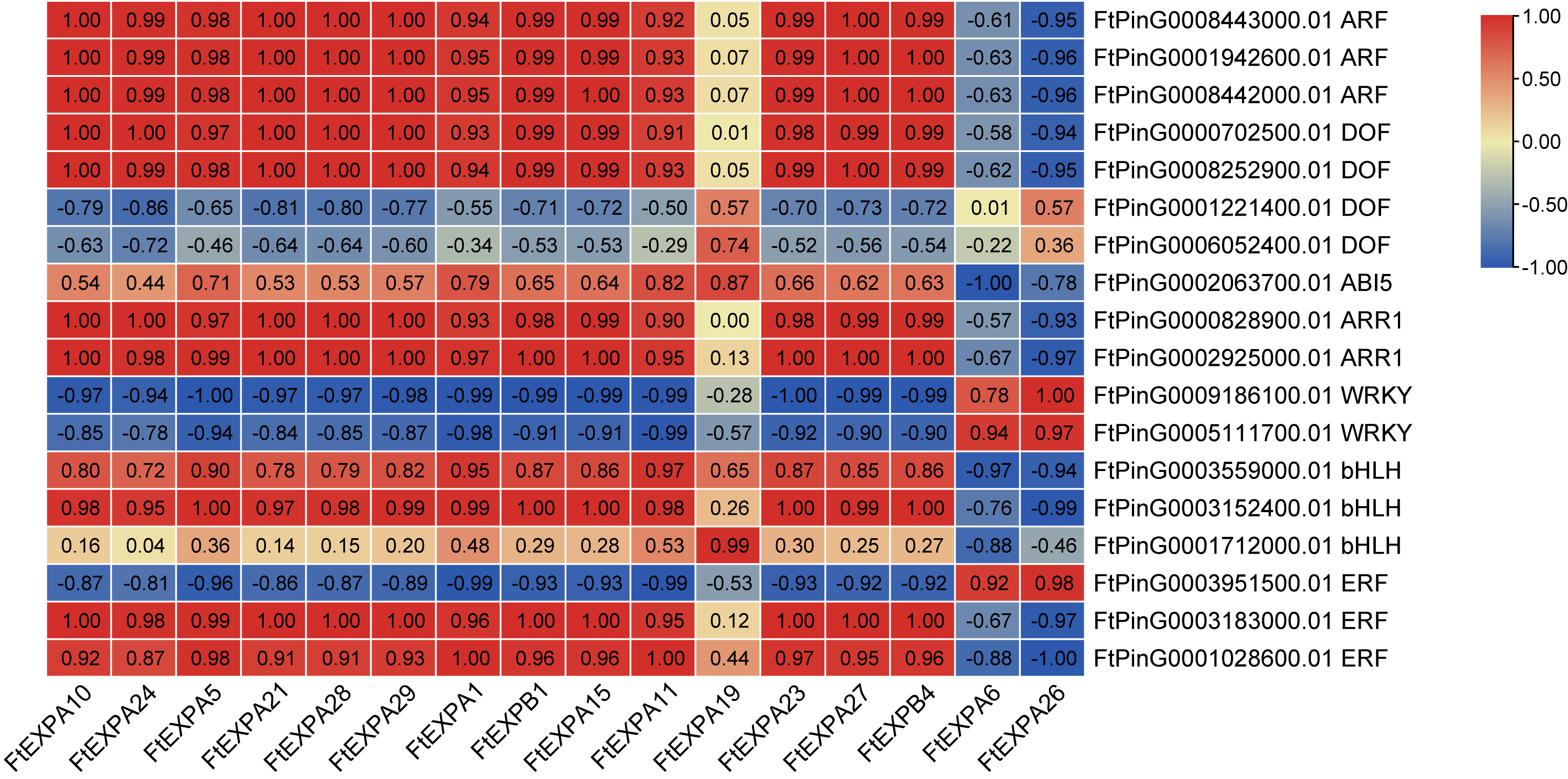


**Supplementary Figure 11.** The correlation between differentially expressed expansin genes and part of the transcription factors. The values in the heatmap indicate the correlation coefficient by the Pearson correlation analysis.


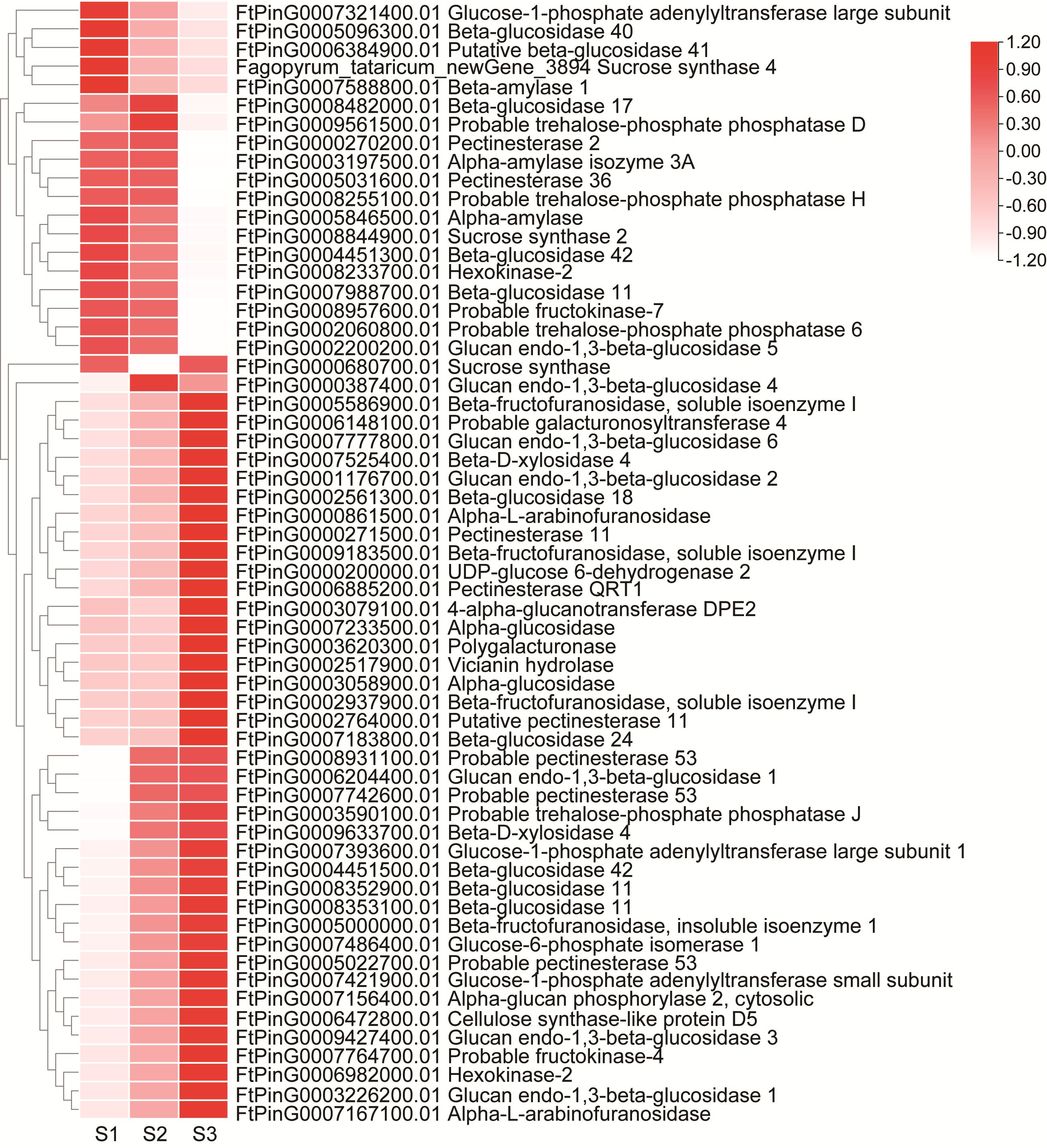


**Supplementary Figure 12.** Heatmap illustration of DEGs (*p* <0.01, FC>4) involved in starch and sucrose metabolism.


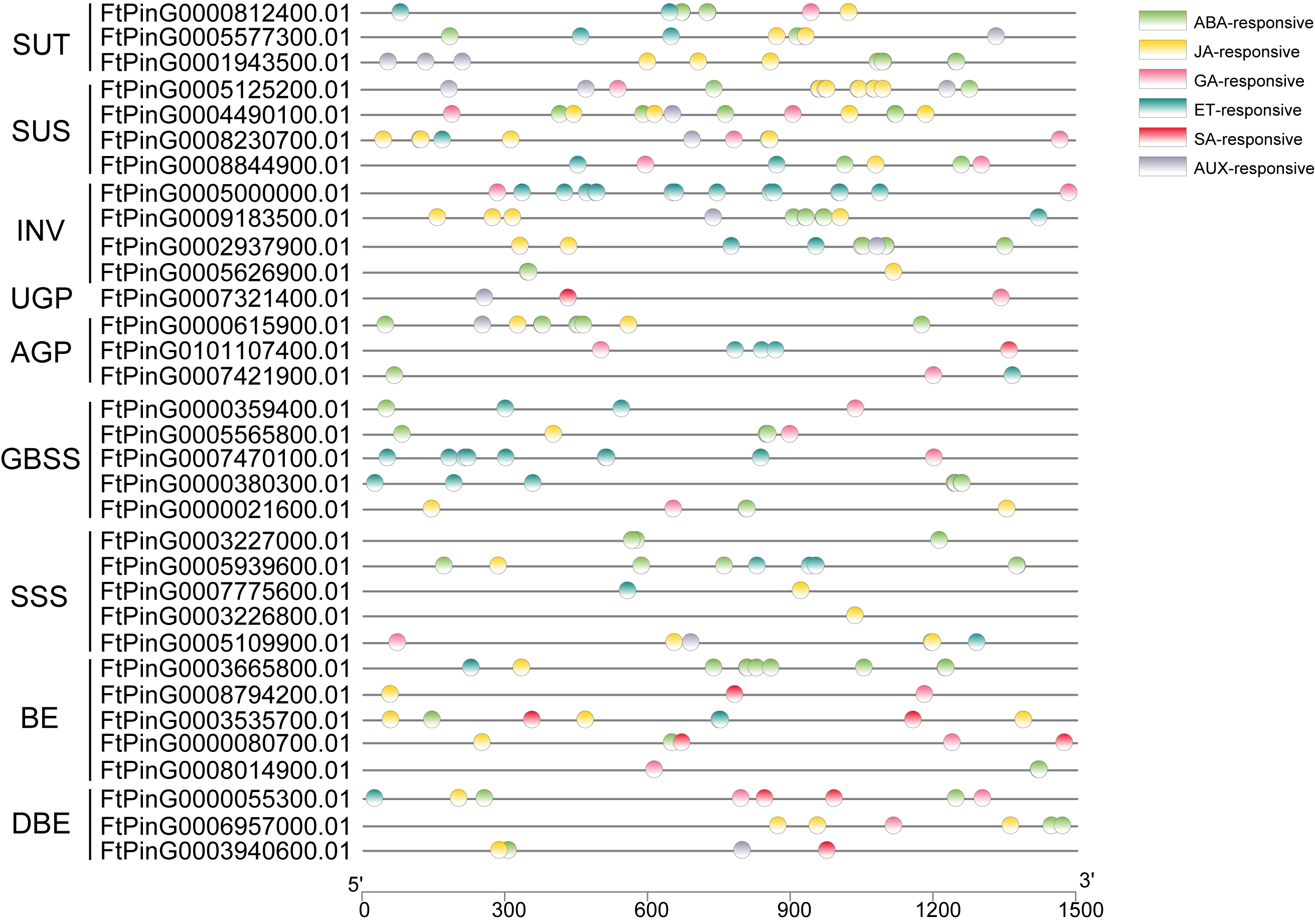


**Supplementary Figure 13.** Illustration of hormone-responsive *cis*-acting elements in the promoter region of differentially expressed structural genes involved in starch biosynthesis.


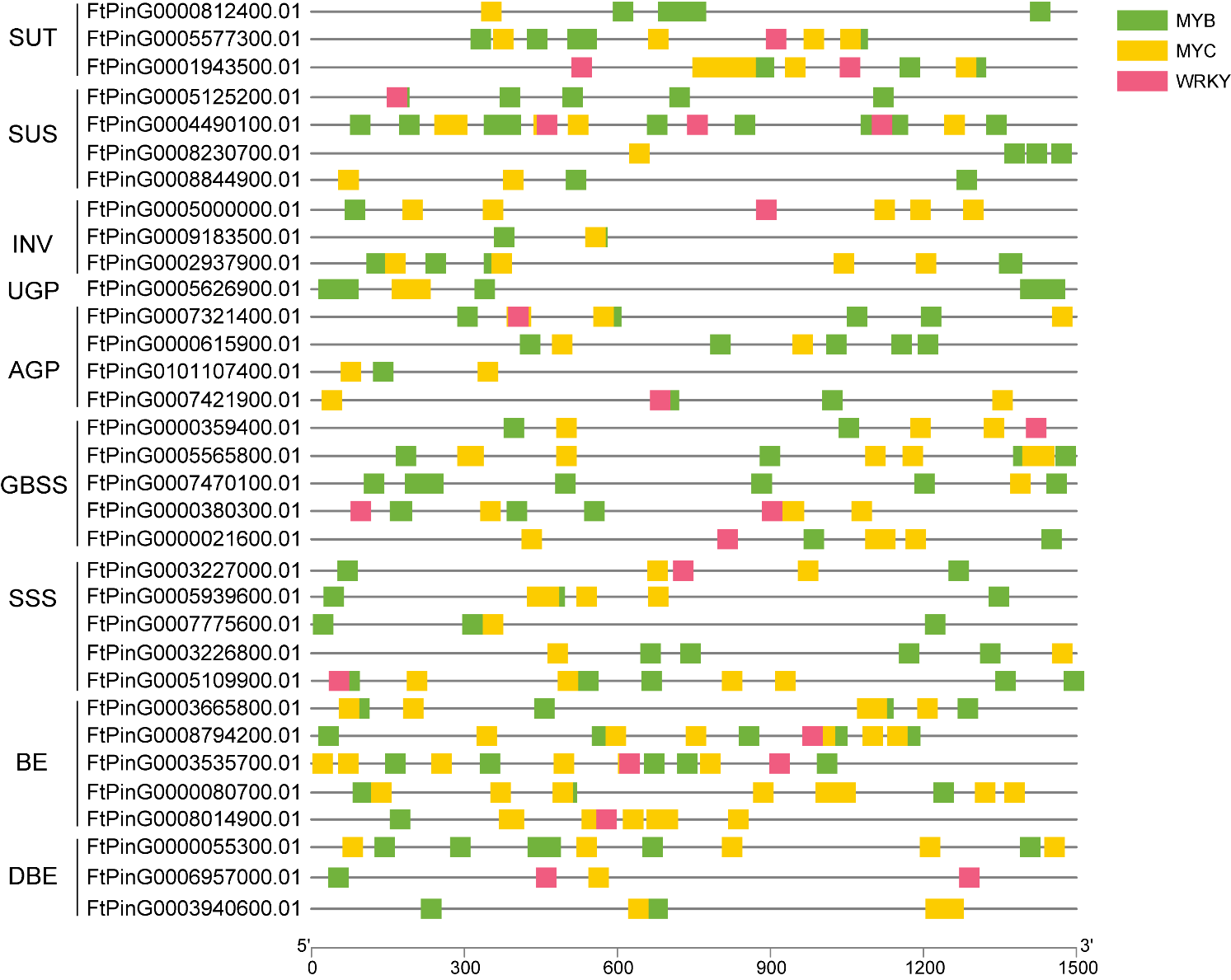


**Supplementary Figure 14.** Illustration of the MYB-, MYC-, and WRKY-binding *cis*-acting elements in the promoter region of differentially expressed structural genes involved in starch biosynthesis.
